# Nutritional Evaluation of Commercial Dog and Cat Foods Based on Key Nutrient Requirements

**DOI:** 10.3390/ani16060909

**Published:** 2026-03-13

**Authors:** Hyun-Woo Cho, Min Young Lee, Woo-Do Lee, Sang-Yeob Lee, Ki Hyun Kim, Kyoung-Min So

**Affiliations:** 1 Animal Welfare Division, National Institute of Animal Science, Rural Development Administration, Wanju 55365, Republic of Korea; jhwoo3856@korea.kr (H.-W.C.); mylee1231@korea.kr (M.Y.L.); woodo92@korea.kr (W.-D.L.); sangnext@korea.kr (S.-Y.L.); 2 Academic-Industrial Cooperation Organization, Sunchon National University, Suncheon 57922, Republic of Korea; kihyun@scnu.ac.kr

**Keywords:** pet food, nutritional adequacy, life stage nutrition, guaranteed analysis, feed regulation

## Abstract

This study evaluated the nutritional adequacy of commercial dog and cat foods in South Korea by comparing analytically determined nutrient contents with recommended nutrient levels of the National Institute of Animal Science (NIAS) and the Association of American Feed Control Officials (AAFCO). A total of 96 pet food products for puppies (*n* = 50), adult dogs (*n* = 18), kittens (*n* = 17), and adult cats (*n* = 11) were collected. Nutrients, including crude protein, crude fat, essential amino acids, essential fatty acids, major minerals, and selected trace nutrients, were analyzed using accredited methods stipulated by the Korean Feed Control Act and compared with NIAS and AAFCO recommendations. Most adult dog and cat diets met recommended nutrient levels; however, deficiencies were identified in diets intended for growth. Puppy foods showed inadequate levels of eicosapentaenoic and docosahexaenoic acids (EPA + DHA, 72%), calcium (22%), and phosphorus (42%), as well as imbalanced calcium-to-phosphorus ratios (12%). In kitten diets, insufficient EPA + DHA (41.2%) and taurine (11.8%) were observed. In contrast, 82.3% of products met label-declared guaranteed analysis values for seven mandatory nutrients. These results provide baseline information on the nutritional adequacy and labeling compliance of pet foods across different life stages.

## Simple Summary

The nutritional quality of dog and cat foods directly influences the health of companion animals across different life stages. In this study, commercially available dog and cat foods in South Korea were analyzed to evaluate their compliance with recommended nutrient levels. While most diets formulated for adult animals met these recommendations, some diets intended for growth did not provide adequate levels of key essential nutrients. In addition, discrepancies between label-declared values and measured nutrient contents were identified in several products. These findings provide practical reference data on the nutritional quality of commercial pet foods and may support improvements in feed formulation, quality control, and nutrition-based labeling systems.

## 1. Introduction

Companion animals have become an important part of people’s lives worldwide, and the pet industry has continued to expand in response to this trend [1,2]. The ownership of dogs and cats has steadily increased, with over 26% of Korean households keeping companion animals [3]. These social changes have stimulated industrial and academic efforts to improve animal health and welfare, with pet food representing the most central sector [4]. Proper nutrition is a key determinant of health and well-being in companion animals, and staple pet food must be formulated to provide balanced nutrient intake without excess or deficiency according to life-stage requirements [5,6]. In particular, inadequate or imbalanced levels of essential nutrients during growth may adversely affect skeletal development, metabolic regulation, and long-term health outcomes. Therefore, whether pet foods provide a complete and balanced nutrient profile is a fundamental criterion for evaluating product quality and safety [7,8].

Nutrient requirements and the quality of dog and cat foods are regulated internationally by governing bodies, such as the Association of American Feed Control Officials (AAFCO) and the European Pet Food Industry Federation (FEDIAF), whose guidelines are widely applied in the pet food industry [6,9]. These guidelines specify the recommended nutrient levels according to the physiological status (e.g., growth, adult maintenance, pregnancy, and activity level) to ensure the nutritional adequacy of commercial products [10]. These products may be labeled as “complete and balanced,” and feed manufacturers are required to declare guaranteed analysis values, which include minimum contents for crude protein and crude fat and maximum contents for crude fiber and moisture on product labels. [11].

In Korea, there is a need to establish national standards, which has led the National Institute of Animal Science (NIAS) to publish Korean nutritional guidelines for pet foods [12]. In addition, revisions to the subordinate regulations of the Feed Management Act, including updated standards, specifications, and labeling requirements, have strengthened the regulatory environment of the domestic pet food market [13]. Accordingly, evaluating whether commercial pet foods distributed in Korea comply with these nutritional standards is a timely issue for proactive regulatory adaptation and to support improvements in product quality within the domestic pet food industry [14]. Despite these developments, systematic evaluations of the nutritional adequacy and labeling compliance of commercial pet foods distributed in Korea remain limited. In particular, there is insufficient evidence comparing analytically determined nutrient concentrations with both national (NIAS) and international (AAFCO) standards across different life stage diets.

Therefore, the present study analyzed major nutrients—essential amino acids, essential fatty acids, minerals, and selected trace nutrients—covering a total of 30 items in 96 commercial dog and cat foods (puppy, adult dog, kitten, and adult cat diets) in Korea. To our knowledge, this study represents the first large-scale analysis of the nutritional composition of commercially available pet foods in Korea. This study aimed to provide objective, evidence-based information on the nutritional adequacy of commercially available dog and cat foods in Korea in terms of compliance with established NIAS and AAFCO nutrient standards, thereby supporting the advancement of nutritional formulation practices and quality assurance strategies in the pet food industry by employing the official analytical methods specified in the Feed Management Act and comparing the measured nutrient concentrations with the minimum recommended nutrient levels determined by NIAS and AAFCO.

## 2. Materials and Methods

### 2.1. Sample Collection

A total of 96 commercial dry and wet dog and cat foods, distributed in South Korea in 2023, were purchased from retail pet supply stores and online markets and analyzed as commercially available products. The unequal number of products between groups was due to differences in product availability in the domestic market at the time of sampling. Products were selected based on their commercial availability at the time of purchase, without stratification by price, brand popularity, or ingredient composition. The samples were classified by life stage (puppy, adult dog, kitten, or adult cat) and food type (dry and wet), as presented in Table 1. Classification was based on the information provided on the product labels, which were visually inspected and photographed to verify all legally required labeling components. Products labeled as ‘all life stages’ were classified as puppy or kitten diets, while only those explicitly labeled for adults were categorized as adult diets. Samples were procured in two separate batches to accommodate product availability and stored in strict accordance with the storage instructions indicated on product packaging until analysis (e.g., dry foods at room temperature, wet foods refrigerated). All products were stored for less than one week prior to analysis and were opened simultaneously for laboratory submission. All samples were analyzed within their declared shelf life. The storage conditions adhered to the label instructions during transport to an accredited testing facility (Feed Technology Laboratory, Korea Feed Association).

### 2.2. Nutrient and Mineral Determination

A total of 29 nutrient parameters were analyzed for dogs (puppy and adult), whereas 30 parameters were analyzed for cats (kitten and adult), with taurine included as an additional essential nutrient for cats. The 29 nutrient parameters were selected to align with regulatory requirements (NIAS and AAFCO) and to cover nutrients with established physiological importance across different life stages of dogs and cats. The following nutrient categories were quantified: crude protein (CP) and 12 essential amino acids (arginine, histidine, isoleucine, leucine, lysine, methionine, methionine + cystine, phenylalanine, phenylalanine + tyrosine, threonine, tryptophan, and valine). Ether extract (EE) and four essential fatty acids (linoleic acid, α-linolenic acid, eicosapentaenoic and docosahexaenoic acids [EPA + DHA]). Seven major minerals (calcium, phosphorus, Ca:P ratio, potassium [K], sodium [Na], magnesium [Mg], and chloride [Cl–]) were quantified. Five trace minerals (copper [Cu], iron [Fe], manganese [Mn], selenium [Se], and zinc [Zn]) were quantified. All analyses were performed using the official analytical methods specified in the subordinate regulations of the Feed Management Act of the Republic of Korea, and detailed procedures can be found in the official regulations. CP content was quantified using the Dumas combustion method (automatic nitrogen analyzer). Amino acids were determined by ion-exchange chromatography with ninhydrin detection. The ether extract (EE; crude fat) was analyzed using the filter bag technique (acid hydrolysis followed by ether extraction), and fatty acids were quantified using Method II of the general fatty acid test procedure. Major minerals (Ca, P, K, Na, and Mg) and trace elements (Mn, Fe, Zn, Cu, and Se) were quantified using inductively coupled plasma–optical emission spectrometry (ICP-OES), and chloride (Cl–) was measured using the Quantab^®^ chloride titration method. The moisture content was analyzed using the forced drying loss by heating method. Crude fiber (CF) content was measured using the filter bag method, and crude ash content was measured using the standard procedures outlined in the official feed analysis guidelines.

### 2.3. Calculation of Metabolizable Energy

All nutrient values used for metabolizable energy calculations and subsequent adequacy assessment were expressed on a dry matter basis to ensure consistent comparison between dry and wet products. The metabolizable energy (ME) was calculated for each product based on energy density equations recommended by the National Research Council (NRC) and the NIAS nutritional guidelines, which are standard for estimating energy content in dog and cat foods [7,12]. The following equations were used:

Gross energy (GE; kcal) = (5.7 × crude protein [%]) + (9.4 × crude fat [%]) + [4.1 × (nitrogen-free extract [%] + crude fiber [%])]

Energy digestibility_Dogs = 91.2 − (1.43 × crude fiber [% DM])

Energy digestibility_Cats = 87.9 − (0.88 × crude fiber [% DM])

Digestible energy (DE, kcal) = [GE (kcal) × digestibility]/100

Metabolizable energy_Dogs (kcal) = DE (kcal) − (1.04 × CP [%])

Metabolizable energy_Cats (kcal) = DE (kcal) − (0.77 × CP [%])

### 2.4. Nutritional Adequacy Assessment

The measured nutrient contents was converted to grams per 1000 kcal ME and compared with the minimum recommended nutrient levels specified by life stage according to the NIAS nutritional guidelines. The number and proportion of products below the recommended values were calculated to evaluate the nutritional adequacy. For nutrients with separate recommendations for early and late growth stages, a higher minimum requirement (early growth) was applied as the evaluation criterion. The NIAS feeding standards were developed with reference to both AAFCO and FEDIAF nutrient guidelines. Given the substantial similarity between NIAS and FEDIAF recommendations for many nutrients, the present study focused on comparison with AAFCO standards.

### 2.5. Compliance Assessment of Guaranteed Analysis

All products listed guaranteed analysis values for seven mandatory nutrients (CP, EE, CF, crude ash, Ca, P, and moisture content), as required by the Korean feed labeling regulations. The guaranteed analysis values were compiled and compared with the measured nutrient concentrations. All nutrients were evaluated on a dry-matter (DM) basis, and for moisture content, compliance was assessed using the 0.66% adjustment formula specified in the Korean Feed Management Act to account for analytical tolerances [15]. Minimum guaranteed nutrients (CP, EE, Ca, and P) were evaluated for compliance when the measured values fell below the declared guarantees, whereas maximum guaranteed nutrients (CF, crude ash, and moisture content) were evaluated when the measured values exceeded the declared guarantees.

### 2.6. Statistical Analysis

Descriptive statistics, including the median, standard deviation (SD), and minimum and maximum values, were used to summarize the nutrient concentrations by group after listing the quantified nutrient values for each group. Median values were additionally presented to provide a robust measure of central tendency given the variability among commercially available products. Compliance values (ratio of measured to guaranteed nutrient content) are expressed as percentages (%), and the mean ± SD and nutrient-specific minimum and maximum ranges are presented to illustrate the overall distribution patterns.

## 3. Results

### 3.1. Analyzed Information of Commercial Pet Foods

The ingredients and product information of the 96 commercial pet foods analyzed in this study are summarized in Table S1. The products were categorized as dog (*n* = 68) or cat foods (*n* = 28). Puppy and kitten foods included products explicitly labeled for growth and all life stage diets that were not specifically designated for adult or senior animals. Only dry and wet staple foods intended for daily feeding were included; functional diets and treats were excluded. Six homemade style products were included (two for puppies, two for adult dogs, one for kittens, and one for adult cats). Three canned products were included in the kitten food category, and all adult cat foods were dry diets. All the analyzed products were commercially available at the time of purchase. Sample quantities were purchased based on analytical requirements regardless of package size, and all products were stored and analyzed within the same timeframe to minimize storage-related variation.

### 3.2. Nutritional Adequacy of Commercial Dog Foods

The nutrient composition and adequacy of the 50 puppy foods relative to the recommended nutrient levels established by NIAS and AAFCO are presented in Table 2. Among these products, 40 were dry foods and 10 were wet foods. The median CP content was 78.9 g/1000 kcal (range, 44.90–232.71), substantially exceeding the recommended level of 56.3 g/1000 kcal, with 94% of products meeting the recommended value. The median concentrations of the essential amino acids arginine, lysine, and valine were 4.87, 4.24, and 3.54 g/1000 kcal, respectively, and all products met the recommended levels for these amino acids. The median EE content was 38.14 g/1000 kcal (range, 4.57–70.45), exceeding the NIAS recommended level of 21.25 g/1000 kcal, with 92% of products meeting the recommendation. Linoleic acid and α-linolenic acid also met the recommended levels in 92% of the products. In contrast, the median EPA + DHA content was 0 g/1000 kcal, with a mean of 0.22 g/1000 kcal. Fourteen products contained the recommended level of EPA + DHA (0.1 g/1000 kcal), whereas 72% of the products showed no detectable EPA + DHA levels. Median Ca and P concentrations were 3.32 and 1.68 g/1000 kcal, respectively, meeting NIAS recommended levels, with adequacy rates of 78% for Ca and 58% for P. However, four products exceeded the recommended upper limit for Ca (6.25 g/1000 kcal), with values of 6.54, 6.91, 7.01 and 7.51 g/1000 kcal. The Ca:P ratio met the recommended range (1:1–2:1) in 88% of the products. These nutrients are considered essential for proper skeletal and developmental processes during growth. Among the trace elements, Se met the recommended levels in 40% of the products, whereas the adequacy rates for Zn, Cu, Fe, and Mn were 76, 80, 90, and 94%, respectively.

The nutrient composition and adequacy of 18 adult dog foods are presented in Table 3. Fourteen products were dry foods and four were wet foods. Overall, adult dog foods demonstrated high compliance with recommended nutrient levels, and deviations were limited to a small number of products. The median crude protein content was 75.45 g/1000 kcal (range, 54.95–188.20), exceeding the recommended level of 45.00 g/1000 kcal in all products. All essential amino acids met recommended levels except for tryptophan, which was slightly below the recommended level (0.40 g/1000 kcal) in one product (0.36 g/1000 kcal). All products met the recommended EE level of 13.75 g/1000 kcal (range, 18.93–70.19), and all but one product (2.61 g/1000 kcal) met the recommended linoleic acid level (2.80 g/1000 kcal). All the products met both the minimum and maximum recommended levels for Ca and P. However, two products exhibited Ca:P ratios outside the recommended ranges (0.64:1 and 2.2:1). The adequacy rates for Cl– and Mg were 73.3% and 94.4%, respectively. Among the trace elements, Cu met recommended levels in 94.4% of products, whereas Se met the NIAS recommended level of 45 µg/1000 kcal in 27.8% of products.

### 3.3. Nutritional Adequacy of Commercial Cat Foods

Among 17 kitten foods (14 dry and 3 wet), the median CP content was 90.90 g/1000 kcal (range, 80.83–170.35), exceeding the NIAS recommended level of 70.00 g/1000 kcal (Table 4). All products met the recommended CP levels. For methionine, methionine + cystine, and tryptophan, some products failed to meet the AAFCO recommendations, which are higher than the NIAS values; however, all products met the NIAS recommended levels. For extruded diets, the recommended taurine level (0.25 g/1000 kcal) was not met by two products (0.22 and 0.24 g/1000 kcal). Among canned diets, although only three products were analyzed, two products contained taurine levels of 0.30 and 0.46 g/1000 kcal, below the recommended level of 0.5 g/1000 kcal. Given the limited number of canned kitten foods analyzed (*n* = 3), these percentages should be interpreted with caution. The recommended EE level of 22.50 g/1000 kcal was met by all products except one, which contained 16.46 g/1000 kcal (range, 16.46–69.45). All products met recommended levels for linoleic acid and α-linolenic acid, whereas EPA + DHA met the recommended level of 0.03 g/1000 kcal in 58.8% of products. The NIAS recommended levels for calcium and phosphorus are 2.5 and 2.0 g/1000 kcal, respectively, and two products failed to meet both recommendations. For minerals and trace elements, no more than three products failed to meet the recommended levels for any nutrient, except Cl– and Se. Because the NIAS recommended levels for Cl– and Cu are lower than those of AAFCO, one product differed for each nutrient. Se met the recommended level of 75.0 g/1000 kcal in 64.7% of products.

All 11 adult cat foods were dry diets. The median CP content was 89.28 g/1000 kcal (range, 74.30–100.58), and all essential amino acids, including taurine, exceeded recommended levels (Table 5). However, these four products did not meet recommended levels for K or Se. The recommended K level is 1.50 g/1000 kcal, and four products contained 1.33, 1.46, 1.47, and 1.48 g/1000 kcal, respectively. For Se, four products showed non-detectable values (0 µg/1000 kcal) relative to the recommended level of 52.50 µg/1000 kcal, whereas the seven products in which Se was detected in the range of 341.86–590.74 µg/1000 kcal. The presence of non-detectable values may partly reflect analytical limitations associated with trace-level selenium determination.

### 3.4. Product Label Guaranteed Analysis Fulfillment

The compliance between the guaranteed analysis values declared on the product labels and the measured nutrient concentrations is summarized in Table 6. After applying the official analytical methods and permissible analytical tolerances specified by the Feed Management Act, agreement between declared and measured values for the seven mandatory nutrients was confirmed in 82.3% of products. Compliance with declared minimum CP levels was 96.0%, 100%, 88.2%, and 100% for puppy, adult dog, kitten, and adult cat foods, respectively. Compliance with declared minimum ether extract (EE) levels was 96.0%, 100%, 94.0%, and 100%, respectively. For maximum guaranteed nutrients, crude ash levels complied in 96.0%, 100%, 94.0%, and 91.0% of puppy, adult dog, kitten, and adult cat foods, respectively. Crude fiber (CF) levels complied in 100%, 94.1%, and 90.9% of dog, kitten, and adult cat foods, respectively. For Ca, P, and moisture content, compliance rates were 94.0% for Ca, P, and moisture in puppy foods; 94.4% for Ca and 100% for P and moisture in adult dog foods; and 100% for Ca, P, and moisture in both kitten and adult cat foods. All 79 dry foods had moisture contents ≤14%, with a median of 7.7% (range, 3.54–14.0%). As summarized in Table 6, deviations from declared values were generally limited and occurred in a small number of products within each life stage category. No deviations in crude protein or ether extract were observed in adult dog and adult cat foods, whereas a few cases were identified in puppy and kitten foods. Overall compliance was slightly lower in growth-stage diets (72.0% in puppy foods and 70.6% in kitten foods) compared with the overall mean compliance rate of 82.3%.

**Table 2:** Nutrient composition of commercial puppy foods (*n* = 50) and comparison with NIAS and AAFCO recommended nutrient levels.

Nutrients (Unit)	Minimum Levels		Median	Range	% Below Minimum (*n*)	
	NIAS	AAFCO			NIAS	AAFCO
Protein (g)	56.30	56.30	78.90	**44.90**–232.71	6.0 (3)	6.0 (3)
Arginine (g)	2.04	2.50	4.87	2.66–14.63	–	–
Histidine (g)	0.98	1.10	1.77	**0.86**–15.24	2.0 (1)	2.0 (1)
Isoleucine (g)	1.63	1.78	2.86	**1.45**–11.07	2.0 (1)	4.0 (2)
Leucine (g)	3.23	3.23	6.08	**2.89**–18.47	2.0 (1)	2.0 (1)
Lysine (g)	2.20	2.25	4.24	2.26–20.48	–	–
Methionine (g)	0.88	0.88	1.97	**0.75**–6.40	4.0 (2)	4.0 (2)
Methionine + Cystine (g)	1.75	1.75	2.82	**1.46**–8.84	4.0 (2)	4.0 (2)
Phenylalanine (g)	1.63	2.08	3.23	**1.61**–9.22	2.0 (1)	2.0 (1)
Phenylalanine + Tyrosine (g)	3.25	3.25	5.33	**2.73**–16.23	2.0 (1)	2.0 (1)
Threonine (g)	2.03	2.60	2.85	**1.61**–10.15	4.0 (2)	28.0 (14)
Tryptophan (g)	0.50	0.50	0.56	**0.33**–2.21	28.0 (14)	28.0 (14)
Valine (g)	1.70	1.70	3.54	1.91–11.62	–	–
Fat (g)	21.25	21.30	38.14	**4.57**–70.45	8.0 (4)	8.0 (4)
Linoleic acid (g)	3.25	3.30	7.22	**0.45**–11.75	8.0 (4)	8.0 (4)
ALA (g)	0.20	0.20	0.81	**0.00**–3.65	8.0 (4)	8.0 (4)
EPA + DHA (g)	0.10	0.10	**0.00**	**0.00**–2.78	72.0 (36)	72.0 (36)
Calcium (g)	2.50	3.00	3.32	**0.12**–7.51	22.0 (11)	32.0 (16)
Phosphorus (g)	2.25	2.50	2.44	**1.14**–4.14	42.0 (21)	54.0 (27)
Ca:P ratio	1:1	1:1	1.44	**0.07**–1.86	12.0 (6)	12.0 (6)
Potassium (g)	1.10	1.50	1.68	**0.94**–4.76	2.0 (1)	22.0 (11)
Sodium (g)	0.55	0.80	0.80	**0.19**–3.18	16.0 (8)	50.0 (25)
Chloride (g)	0.83	1.10	1.10	**0.24**–1.92	28.0 (14)	48.0 (24)
Magnesium (g)	0.10	0.15	0.33	0.15–0.78	–	–
Copper (mg)	2.75	3.10	5.51	**0.33**–20.03	20.0 (10)	20.0 (10)
Iron (mg)	22.00	22.00	88.48	**10.43**–228.40	10.0 (5)	10.0 (5)
Manganese (mg)	1.40	1.80	12.38	**0.43**–39.66	6.0 (3)	6.0 (3)
Selenium (µg)	90.00	90.00	**0.00**	**0.00**–559.78	60.0 (30)	60.0 (30)
Zinc (mg)	25.00	25.00	53.12	**5.42**–130.11	24.0 (12)	24.0 (12)

Values are expressed per 1000 kcal of metabolizable energy (ME). “% below minimum” indicates the percentage (and number) of products with nutrient concentrations below the recommended level. The en dashes (–) indicate that all products met the recommended level. Bold values indicate nutrients below that recommended by the NIAS. Underlined values indicate nutrients levels below that recommended by the AAFCO. NIAS, National Institute of Animal Science; AAFCO, Association of American Feed Control Officials; ALA, α-linolenic acid; EPA, eicosapentaenoic acid; DHA, docosahexaenoic acid.

**Table 3:** Nutrient composition of commercial adult dog foods (*n* = 18) and comparison with NIAS- and AAFCO-recommended nutrient levels.

Nutrients (Unit)	Minimum Levels		Median	Range	% Below Minimum (*n*)	
	NIAS	AAFCO			NIAS	AAFCO
Protein (g)	45.00	45.00	75.45	54.95–188.20	–	–
Arginine (g)	1.28	1.28	4.05	3.46–11.14	–	–
Histidine (g)	0.48	0.48	1.69	1.10–4.71	–	–
Isoleucine (g)	0.95	0.95	2.80	1.74–8.33	–	–
Leucine (g)	1.70	1.70	5.84	3.50–14.40	–	–
Lysine (g)	1.05	1.58	3.61	2.66–15.49	–	–
Methionine (g)	0.83	0.83	1.91	1.03–5.45	–	–
Methionine + Cystine (g)	1.63	1.63	2.78	1.85–7.28	–	–
Phenylalanine (g)	1.13	1.13	3.41	2.16–7.34	–	–
Phenylalanine + Tyrosine (g)	1.85	1.85	5.52	3.66–13.40	–	–
Threonine (g)	1.20	1.20	2.66	1.71–8.60	–	–
Tryptophan (g)	0.40	0.40	0.51	**0.36**–1.38	5.6 (1)	5.6 (1)
Valine (g)	1.23	1.23	3.33	2.24–9.78	–	–
Fat (g)	13.75	13.80	36.09	18.93–70.19	–	–
Linoleic acid (g)	2.80	2.80	7.95	**2.61**–12.05	5.6 (1)	5.6 (1)
ALA (g)	–	–	0.97	0.30–3.89	–	–
EPA + DHA (g)	–	–	0.09	0.00–3.50	–	–
Calcium (g)	1.25	1.25	3.32	1.25–5.19	–	–
Phosphorus (g)	1.00	1.00	2.47	1.36–3.72	–	–
Ca:P ratio	1:1	1:1	1.39	**0.64**–2.20	5.6 (1)	5.6 (1)
Potassium (g)	1.25	1.50	1.81	1.37–2.64	–	–
Sodium (g)	0.20	0.20	0.85	0.32–4.25	–	–
Chloride (g)	0.30	0.30	1.18	**0.16**–3.74	16.7 (3)	16.7 (3)
Magnesium (g)	0.15	0.15	0.35	**0.12**–0.54	5.6 (1)	5.6 (1)
Copper (mg)	1.80	1.83	5.22	**1.40**–19.83	5.6 (1)	5.6 (1)
Iron (mg)	9.00	10.00	82.11	35.95–244.98	–	–
Manganese (mg)	1.25	1.25	15.00	3.50–28.07	–	–
Selenium (µg)	45.00	80.00	**0.00**	**0.00**–478.00	72.2 (13)	72.2 (13)
Zinc (mg)	18.00	20.00	67.08	24.85–120.42	–	–

Values are expressed per 1000 kcal of metabolizable energy (ME). “% below minimum” indicates the percentage (and number) of products with nutrient concentrations below the recommended level. The en dashes (–) indicate that either no recommended nutrient level has been established for that nutrient, or that all products meet the recommended level. Bold values indicate nutrient levels below that recommended by the NIAS. Underlined values indicate nutrient levels below that recommended by the AAFCO. NIAS, National Institute of Animal Science; AAFCO, Association of American Feed Control Officials; ALA, α-linolenic acid; EPA, eicosapentaenoic acid; DHA, docosahexaenoic acid.

**Table 4:** Nutrient composition of commercial kitten foods (*n* = 17) and comparison with NIAS and AAFCO recommended nutrient levels.

Nutrients (Unit)	Minimum Levels		Median	Range	% Below Minimum (*n*)	
	NIAS	AAFCO			NIAS	AAFCO
Protein (g)	70.00	75.00	90.90	80.83–170.35	–	–
Arginine (g)	2.68	3.10	5.53	4.02–9.91	–	–
Histidine (g)	0.83	0.83	1.95	1.57–7.11	–	–
Isoleucine (g)	1.35	1.40	3.26	2.74–6.41	–	–
Leucine (g)	3.20	3.20	6.26	4.89–11.19	–	–
Lysine (g)	2.13	3.00	5.12	3.55–12.00	–	–
Methionine (g)	1.10	1.55	2.08	1.29–3.79	–	11.8 (2)
Methionine + Cystine (g)	2.20	2.75	3.25	2.44–5.28	–	11.8 (2)
Phenylalanine (g)	1.25	1.30	3.64	2.52–6.41	–	–
Phenylalanine + Tyrosine (g)	4.78	4.80	5.78	**4.36**–10.84	5.9 (1)	5.9 (1)
Threonine (g)	1.63	1.83	3.40	2.74–6.76	–	–
Tryptophan (g)	0.40	0.63	0.67	0.46–1.17	–	5.9 (1)
Valine (g)	1.55	1.55	4.03	3.10–7.69	–	–
Taurine-extruded (g)	0.25	0.25	0.58	**0.22**–1.18	14.3 (2)	14.3 (2)
Taurine-canned (g)	0.50	0.50	**0.46**	**0.30**–8.72	66.7 (2)	66.7 (2)
Fat (g)	22.50	22.50	38.17	**16.46**–69.45	5.9 (1)	5.9 (1)
Linoleic acid (g)	1.38	1.40	7.25	1.98–11.80	–	–
ALA (g)	0.05	0.05	0.69	0.20–2.19	–	–
EPA + DHA (g)	0.03	0.03	0.19	**0.00**–6.04	41.2 (7)	41.2 (7)
Calcium (g)	2.50	2.50	4.05	**0.88**–6.85	11.8 (2)	11.8 (2)
Phosphorus (g)	2.00	2.00	2.92	**0.78**–4.23	11.8 (2)	11.8 (2)
Ca:P ratio	–	–	1.34	0.66–1.73	–	–
Potassium (g)	1.50	1.50	1.81	**0.60**–3.42	17.6 (3)	17.6 (3)
Sodium (g)	0.40	0.50	1.24	**0.28**–1.59	5.9 (1)	5.9 (1)
Chloride (g)	0.60	0.75	1.69	0.73–7.93	–	5.9 (1)
Magnesium (g)	0.13	0.20	0.31	**0.06**–0.58	5.9 (1)	5.9 (1)
Copper-extruded (mg)	2.50	3.75	5.46	**0.50**–12.07	14.3 (2)	21.4 (3)
Copper-canned (mg)	2.50	2.10	4.96	**2.10**–5.04	33.3 (1)	–
Iron (mg)	20.00	20.00	70.17	**8.89**–172.36	11.8 (2)	11.8 (2)
Manganese (mg)	1.90	1.90	7.36	**0.44**–28.03	5.9 (1)	5.9 (1)
Selenium (µg)	75.00	75.00	388.99	**0.00**–1923.07	35.3 (6)	35.3 (6)
Zinc (mg)	18.80	18.80	38.84	**5.76**–104.79	11.8 (2)	11.8 (2)

Values are expressed per 1000 kcal of metabolizable energy (ME). “% below minimum” indicates the percentage (and number) of products with nutrient concentrations below the recommended level. The en dashes (–) indicate that either no recommended nutrient level has been established for that nutrient or that all products meet the recommended level. Bold values indicate nutrient levels below that recommended by the NIAS. Underlined values indicate nutrient levels below that recommended by the AAFCO. NIAS, National Institute of Animal Science; AAFCO, Association of American Feed Control Officials; ALA, α-linolenic acid; EPA, eicosapentaenoic acid; DHA, docosahexaenoic acid.

**Table 5:** Nutrient composition of commercial adult cat foods (*n* = 11) and comparison with NIAS and AAFCO recommended nutrient levels.

Nutrients (Unit)	Minimum Levels		Median	Range	% Below Minimum (*n*)	
	NIAS	AAFCO			NIAS	AAFCO
Protein (g)	62.50	65.00	89.28	74.30–100.58	–	–
Arginine (g)	2.50	2.60	5.18	3.54–6.48	–	–
Histidine (g)	0.65	0.78	1.92	1.54–2.16	–	–
Isoleucine (g)	1.08	1.30	3.16	2.51–3.74	–	–
Leucine (g)	2.55	3.10	6.99	4.97–9.46	–	–
Lysine (g)	0.85	2.08	4.70	2.78–6.41	–	–
Methionine (g)	0.43	0.50	1.97	1.52–3.94	–	–
Methionine + Cystine (g)	0.85	1.00	3.04	2.62–5.08	–	–
Phenylalanine (g)	1.00	1.05	3.74	3.20–4.41	–	–
Phenylalanine + Tyrosine (g)	3.83	3.83	6.11	5.06–7.36	–	–
Threonine (g)	1.30	1.83	3.30	2.35–3.60	–	–
Tryptophan (g)	0.33	0.40	0.73	0.51–0.87	–	–
Valine (g)	1.28	1.55	3.97	3.08–4.46	–	–
Taurine-extruded (g)	0.25	0.25	0.66	0.35–2.14	–	–
Fat (g)	22.50	22.50	37.06	32.80–44.32	–	–
Linoleic acid (g)	1.25	1.40	7.28	5.27–9.28	–	–
ALA (g)	–	–	0.75	0.29–3.76	–	–
EPA + DHA (g)	–	–	0.13	0.00–0.59	–	–
Calcium (g)	1.00	1.50	3.84	2.94–6.24	–	–
Phosphorus (g)	0.64	1.25	2.88	2.08–3.94	–	–
Ca:P ratio	–	–	1.44	1.11–1.79	–	–
Potassium (g)	1.50	1.50	1.62	**1.33**–2.34	36.4 (4)	36.4 (4)
Sodium (g)	0.19	0.50	1.21	0.90–1.47	–	–
Chloride (g)	0.29	0.75	1.61	0.86–2.52	–	–
Magnesium (g)	0.10	0.10	0.32	0.24–0.63	–	–
Copper-extruded (mg)	1.25	1.25	5.77	4.67–12.35	–	–
Iron (mg)	20.00	20.00	82.50	52.84–132.22	–	–
Manganese (mg)	1.25	1.90	17.00	4.89–26.23	–	–
Selenium (µg)	52.50	75.00	379.53	**0.00**–590.74	36.4 (4)	36.4 (4)
Zinc (mg)	18.80	18.80	57.85	25.06–97.58	–	–

Values are expressed per 1000 kcal of metabolizable energy (ME). “% below minimum” indicates the percentage (and number) of products with nutrient concentrations below the recommended level. The en dashes (–) indicate that either no recommended nutrient level has been established for that nutrient, or that all products meet the recommended level. Bold values indicate nutrient levels below that recommended by the NIAS. Underlined values indicate nutrient levels below that recommended by the AAFCO. NIAS, National Institute of Animal Science; AAFCO, Association of American Feed Control Officials; ALA, α-linolenic acid; EPA, eicosapentaenoic acid; DHA, docosahexaenoic acid.

**Table 6:** Verification of label-declared guaranteed analysis for seven nutrients in commercial pet foods based on measured values.

	Puppy		Adult Dog		Kitten		Adult Cat	
	Mean ± SD	Range	Mean ± SD	Range	Mean ± SD	Range	Mean ± SD	Range
CP	116.5 ± 19.37	69–170	116.0 ± 12.38	101–143	124.6 ± 74.97	87–414	110.6 ± 6.48	104–129
EE	146.5 ± 103.21	50–800	133.2 ± 37.35	87–215	120.5 ± 34.11	66–227	122.5 ± 22.49	96–169
CA	77.4 ± 18.70	32–126	76.8 ± 13.52	57–97	84.7 ± 26.95	53–173	81.3 ± 15.91	56–106
CF	48.1 ± 22.12	50–102	56.0 ± 24.55	3–100	165.1 ± 408.91	24–1750	69.4 ± 20.85	37–116
Ca	173.6 ± 102.99	31–636	150.4 ± 51.65	69–309	172.6 ± 60.04	90–317	160.4 ± 30.60	123–219
P	172.3 ± 77.79	47–542	155.6 ± 41.44	93–251	169.4 ± 45.26	115–261	190.8 ± 66.23	115–318
Moisture	72.1 ± 26.44	25–185	76.8 ± 14.51	51–100	60.7 ± 22.13	5–101	63.8 ± 16.63	40–96

Values are expressed as percentages of the label-declared guaranteed values. Data are presented as the mean ± standard deviation (SD) and range. CP, crude protein; EE, crude fat; CA, crude ash; CF, crude fiber; Ca, calcium; P, phosphorus.

## 4. Discussion

The quality and nutritional adequacy of pet foods are major considerations for consumers [16,17]. Although extensive nutritional information and regulatory standards are available, the direct evaluation of nutrient content through laboratory analysis remains the most fundamental approach for assessing product quality [18]. To our knowledge, this study is the first comprehensive evaluation of the nutrient composition of 96 commercially available dog and cat foods in Korea, analyzing 29 nutrients for dogs and 30 nutrients for cats––excluding certain nutrients such as iodine, choline, and vitamins––and quantitatively comparing the results with the recommended nutrient levels established by the NIAS and AAFCO.

With the exception of puppy and kitten foods, which are formulated for life stages that have increased nutrient requirements, such as growth or reproduction, most adult dog and cat foods met the NIAS recommended nutrient levels for nearly all analyzed nutrients. In contrast, among puppy foods, a substantial proportion of products showed insufficient EPA + DHA levels and essential omega-3 fatty acids, which play critical roles in neurological development. EPA and DHA are key structural components of the brain and retina and are essential for cognitive and visual development in puppies, as well as for the modulation of inflammatory responses [19,20,21,22]. Since EPA and DHA are sensitive to heat, it is necessary to account for potential degradation or loss during the manufacturing process. In addition, inadequacies in Ca and P, and imbalances in the Ca:P ratio were observed in puppy foods. The optimal Ca:P ratio ranges from 1:1 to 2:1, and deviations from this range can impair mineral bioavailability, potentially leading to abnormal skeletal development and orthopedic diseases during growth [23,24]. Therefore, energy-dense puppy diets must ensure not only adequate absolute amounts of Ca and P but also the precise maintenance of their optimal balance.

Trace elements such as Cu, Fe, and Zn are also essential for normal growth, immune system development, and enzymatic activity in puppies. Inadequate intake of these micronutrients may compromise growth potential and immune competence [25,26,27]. Consequently, for nutrients with established minimum requirements, formulation strategies that incorporate appropriate safety margins while considering bioavailability are recommended to ensure adequacy without unnecessary excess [28]. In kitten foods, inadequacies were identified for EPA + DHA and taurine. Taurine is an essential amino acid for cats, and deficiency can result in dilated cardiomyopathy (DCM) and central retinal degeneration (CRD), making adequate taurine inclusion a critical determinant of feline diet quality [29,30,31]. Taurine is heat-labile and is particularly susceptible to losses during high temperature, long duration processes, such as the sterilization of canned diets [32]. This highlights the need for further data on taurine stability during formulation, storage, and processing, as well as technological improvements to ensure taurine stability and consistent final concentrations. Adult dog foods generally exhibited high rates of compliance with the recommended nutrient levels; however, one product exhibited a Ca:P ratio outside the recommended range. Although adult animals do not undergo the rapid skeletal changes observed during growth, the long-term consumption of diets with imbalanced Ca:P ratios may affect bone metabolism or renal function [23,33]. Such imbalances may not produce overt short-term clinical signs but warrant careful consideration with prolonged feeding. Among adult cat foods, K showed the highest inadequacy rate, although the absolute deviations from the recommended levels were modest. K is a major electrolyte essential for maintaining muscle and nerve function and intracellular fluid balance in cats. Considering the high prevalence of chronic kidney disease (CKD) in cats, K deficiency (hypokalemia) may exacerbate renal dysfunction and muscle weakness [34,35]. However, the low K levels observed in this study were limited to specific products, and their biological significance may depend on feeding amounts and long-term intake. Thus, the interpretation of these findings should be complemented by studies addressing bioavailability and clinical outcomes. Notably, Se exhibited the highest inadequacy rates across all diet groups (puppy, 60.0%; adult dog, 72.2%; kitten, 35.3%; and adult cat, 36.4%), with non-detectable values observed below the lowest detected concentration of 342 µg/1000 kcal. Given the trace level presence of Se in pet foods, the possibility of analytical limitations, including limits of detection (LOD) and quantification (LOQ), as well as influences from sample preparation and measurement methods, cannot be excluded. Se determination is highly method dependent, with substantial variation in sensitivity depending on the analytical techniques, such as ICP-OES, inductively coupled plasma–mass spectrometry (ICP-MS), and hydride generation atomic absorption spectrometry (HG-AAS), as well as sample pretreatment procedures [36]. High sensitivity ICP-MS platforms, particularly when combined with appropriate pretreatment and concentration steps, have been reported to be more suitable for ultra-trace Se quantification, whereas ICP-OES generally exhibits higher detection limits, which may restrict accurate quantification at low concentrations [36,37]. Moreover, the use of HPLC-ICP-MS or ICP-MS following enhanced pretreatment has been recommended to improve Se quantification by lowering LOQ values [38,39]. These findings suggest that beyond adjusting supplementation levels, greater use of bioavailable organic Se sources and adoption of standardized high-sensitivity analytical methods across laboratories are warranted [40]. Therefore, non-detectable selenium values observed in this study should be interpreted with caution and may not necessarily indicate true nutritional deficiency in the evaluated products.

The recommended nutrient levels, particularly for proteins, minerals, and essential fatty acids, are generally higher in puppy and kitten foods than in adult dog and cat foods to support rapid growth and development [6]. In this study, the overall nutrient adequacy was lower in puppy and kitten foods than in adult foods. These findings indicate that commercially available growth diets may face greater challenges in consistently meeting the elevated nutrient requirements associated with early life stages. Interestingly, when the nutrient contents of the growth diets were reassessed against adult recommended levels, the adequacy rates improved substantially. This finding suggests that many commercially available growth diets struggle to consistently meet the elevated nutrient requirements during the growth stage. Therefore, the development of growth diets should incorporate clearer criteria for life stage-specific nutrient requirements and growth completion benchmarks.

Several methodological limitations of the present study should be considered when interpreting these findings, as discussed below. The NIAS feeding standards provide nutritional guidelines tailored to domestic conditions in Korea, complementing international standards. Compared with AAFCO, the NIAS tends to adopt less conservative recommended levels or different maximum tolerable limits for certain nutrients [6,12], potentially reflecting the ingredient characteristics commonly used in Korean or domestic research data. However, differences between NIAS and AAFCO recommendations may influence formulation targets and regulatory interpretation across markets, highlighting the importance of harmonized nutritional evaluation approaches [6]. Accordingly, domestic manufacturers should not only align formulations with NIAS standards but also strengthen analytical standardization and quality assurance systems. A limitation of this study was that not all age-specific nutrients listed in the feeding standards were analyzed. In particular, some nutrients, such as vitamins, were excluded because of technical constraints associated with quantitative analysis [41]. Nevertheless, by quantitatively demonstrating the nutritional adequacy of commercially available pet foods, this study provides foundational data to support improvements in ingredient formulations, nutrient fortification strategies, and quality control. These findings provides objective evidence to support appropriate nutrition across life stages and enhance the overall nutritional quality of pet foods.

## 5. Conclusions

The results of this study indicate that while many commercial pet foods available in Korea generally align with established nutrient recommendations, variability remains in compliance with declared nutritional standards. In particular, growth-stage diets showed comparatively greater challenges in consistently meeting elevated nutrient requirements. Objective nutrient analysis highlights areas where formulation accuracy and labeling consistency could be improved to ensure nutritionally complete and balanced diets. Although certain nutrients could not be evaluated because of analytical limitations, the present findings provide representative insights into the nutritional adequacy of commercially available products. These findings provide practical reference data to support improvements in pet food formulation, strengthen quality control practices, and facilitate regulatory compliance within the commercial pet food industry.

## Figures and Tables

**Table 1:** Distribution of commercial pet food samples (*n* = 96) included in the analysis.

Group	Diet Form		Total
	Dry	Wet	
Puppy	40	10	50
Adult dog	14	4	18
Kitten	14	3	17
Adult cat	11	0	11

## Data Availability

Upon reasonable request, the datasets of this study can be available from the corresponding author.

## Supplementary Materials

The following supporting information can be downloaded at https://www.mdpi.com/article/10.3390/ani16060909/s1, Table S1: Ingredient lists of commercial pet foods included in this study.
