# Supplementary material for: Nutritional Evaluation of Commercial Dog and Cat Foods Based on Key Nutrient Requirements
Source: Animals (Basel). 2026 Mar 13;16(6):909. doi: 10.3390/ani16060909 (PMC13023309; doi:10.3390/ani16060909)
Supplement: Supplementary file 1 [file animals-16-00909-s001.zip › animals-4151924-supplementary.pdf]

Supplementary Table S1. Ingredient lists of commercial pet foods included in this study.

Product	Ingredients list	Description
Diet 1	Animal Protein Premix (Beef, Salmon, White Fish), Rice, Maize Gluten, Brown Rice, Corn, Oats, Refined Chicken Oil, Barley, Sesame Meal, Natural Flavoring, Flaxseed, Sunflower Seed, Beat Pulp, Mung Bean, Buckwheat, Sweet Potato, Maifanite, Carrot, Pumpkin, Sunflower Meal, Vitamin Premix (Vitamin A, D3, E, B1, B2, B5, B6, Folic Acid, Niacin, Biotin), Mineral Premix (Iron, Copper, Zinc, Selenium, Manganese, Cobalt, Iodine), Choline Chloride, Calcium Carbonate, Rosemary Extract, Dicalcium Phosphate, L-Lysine Sulfate, Methionine, Salt, Vitamin C, Yucca Extract	All Life Stages (Dog)
Diet 2	Hydrolyzed Lamb, Mung Bean, Sunflower Seed Powder, Brown Rice, Whole Barley, Peas, Chicken Fat, Millet, Sweet Potato, Whole Flaxseed, Chicken Meal, Fish Oil, Vitamin Premix, Mineral Premix, Yucca Schidigera Extract, Oats, Sea Salt, Fructooligosaccharide, DL-Methionine, Hydrolyzed Green Lipped Mussel Complex (Source of Chondroitin and Collagen), Red Ginseng, Inulin, Glucosamine, Ascorbic Acid, Dicalcium Phosphate, Beneficial Bacteria Blend, Sea Buckthorn Berry, Evening Primrose Seed, Coriander, Carrot, Spinach, Dried Probiotics Powder	All Life Stages (Dog)
Diet 3	Chicken (Fresh Chicken Meat, Dried Powder [Meat, Liver, Heart, Intestine], Hydrolyzed Protein), Freeze-Dried Duck, Whole Corn, Whole Oatmeal, Rice, Chicken Fat, Broccoli, Apple, Beet, Green Lipped Mussel, Raw Manuka Honey (UMF+5), Turmeric, Peppermint, Psyllium Husk, Beet Pulp, Flaxseed, Brewer's Yeast, L-Lysine, DL-Methionine, L-Threonine, Fructooligosaccharide (FOS), Mannan Oligosaccharide (MOS), Glucosamine, Yucca Extract, Cellulose, Chelated Minerals (Iron, Copper, Zinc, Selenium, Manganese, Cobalt), Natural Antioxidants (Tocopherols, Rosemary Oil), Vitamin Supplement (Vitamin A, D, E, K, B1, B2, B6, B5, Biotin [B8], Folic Acid [B9], Niacin [B3], B12, C), Choline Chloride, Potassium Chloride, Lactobacillus plantarum, Bacillus subtilis, Enzyme (Protease)	All Life Stages (Dog)
Diet 4	Hydrolyzed Salmon, Hydrolyzed Herring, Barley, Rice, Oats, Sunflower Seeds, Flaxseed, Perilla Seeds, Millet, Sweet Potato, Potato, Maifan Stone, Soybean, Herbal Extracts (Licorice, Talc, Ginger, Scutellaria, Platycodon, Gypsum, Rhubarb, Glauber's Salt, Ephedra, Peppermint Leaf, Forsythia, Peony, Angelica, Siler, Cnidium, Aucklandia, Polygala, Honeysuckle, Epimedium, Atractylodes, Gardenia, Schizonepeta), Fermented Folic Acid Polymer Extract, Vitamin C, Tapioca, Egg-Derived Calcium, Chestnut, L-Lysine Sulfate, DL-Methionine, Chlorella, Choline Powder, Omega-3 & 6, Cinnamon, Rosemary, Bacillus subtilis, Oregano, Taurine, Mannan Oligosaccharide, Yucca Extract, Glucosamine, Beta-Glucan, Vitamin Mix (Vitamins A, AD3, B1, B2, B3, B5, B6, Biotin, Folic Acid, B12, D3, E, K3), Mineral Premix (Iron, Manganese, Copper, Cobalt, Iodine, Selenium, Magnesium), Nucleotides	All Life Stages (Dog)

Diet 5	Duck Meat, Rice Flour, Pea Protein Powder, Potato Starch, Beet Pulp, Tapioca, Chicken Oil, Chicory (Root) Pulp, Dried Pollack Powder, Sweet Potato, Hydrolyzed Protein (Liquid), Pumpkin, Hydrolyzed Salmon Protein Powder, Fructooligosaccharide, Seaweed (Kelp Meal), Hydrolyzed Protein (Powder), Omega-3, Salmon Oil, Algal DHA Oil, Collagen	All Life Stages (Dog)
Diet 6	Corn, Wheat, Wheat Bran, Soybean Meal, Meat Meal, Rice Bran, Corn Gluten, Hydrolyzed Protein (Chicken), Animal Fats, Brewer's Yeast, Prebiotics (Mannan-oligosaccharide (MOS), Fructo-oligosaccharide (FOS), Enzyme Complex (Protease), Yucca Extract, L-Lysine, DL-Methionine, L-Threonine, Natural Antioxidants (Tocopherols, Rosemary Oil), Vitamin Premix (Vitamins A, D, E, K, B1, B2, B6, B5, Biotin (B8), Folic Acid (B9), Niacin (B3), B12), Choline Chloride, Mineral Premix (Iron, Copper, Zinc, Selenium, Manganese, Iodine, Cobalt), Potassium Chloride	All Life Stages (Dog)
Diet 7	Hydrolyzed Chicken, Hydrolyzed Salmon, Cassava (Tapioca) Starch, Chicken Oil, Omega-3, Peas, Beet Pulp, Purple Sweet Potato, Brewer's Yeast, Flaxseed, Natural Flavors, Monocalcium Phosphate, Glucosamine, Fructo-oligosaccharide (FOS), Yucca Extract, Lecithin, Vitamin E, Vitamin Premix (Vitamin A, B1, etc.), Mixed Mineral Premix, Seaweed Powder, Refined Salt, L-Lysine, L-Tryptophan, L-Threonine, Choline Chloride, Potassium Chloride, Yeast Culture, Potassium Sorbate, Organic Zinc, Minerals, Lutein, Rosemary Extract, Tocopherols, Collagen	All Life Stages (Dog)
Diet 8	Hydrolyzed Salmon Meat, Brown Rice, Whole Barley, Mung Bean, Sunflower Seed, Sweet Potato, Peas, Potato, Chicken Fat, Whole Flaxseed, Fish Meal, Fish Oil, Dried Krill, Beet Pulp, Yucca Schidigera Extract, Sea Salt, Whole Oats, Fructo-oligosaccharide (FOS), DL-Methionine, Red Ginseng, Hydrolyzed Green-lipped Mussel Complex (source of Chondroitin and Collagen), Sea Buckthorn Fruit, Evening Primrose Seed, Carrot, Coriander, Spinach, Glucosamine, Ascorbic Acid, Monocalcium Phosphate, Beneficial Bacteria Mix, Dried Probiotics, Fucoidan (Seaweed Extract), Vitamins (C, E, A, D3, B1, B2, B6, B12, Biotin, Folic Acid, Pantothenic Acid, Niacin, Choline), Minerals (Iron, Zinc, Copper, Selenium, Iodine, Manganese, Potassium)	All Life Stages (Dog)
Diet 9	Duck Meat, Rice, Corn Gluten Meal, Brown Rice, Oats, Beet Pulp, Refined Chicken Oil, Barley, Flaxseed, Corn, Sesame Meal, Sunflower Meal, Hydrolyzed Protein, Sunflower Seed, Sweet Potato, Mung Bean, Buckwheat, Cellulose, Dicalcium Phosphate, Carrot, Pumpkin, Pumpkin Seed, L-Carnitine, Vitamin Premix, Mineral Premix, Turmeric Powder, Calcium Carbonate, Yucca Extract, Choline Chloride, Cranberry, Probiotics, Vitamin E, Salmon Oil, Fructo-oligosaccharide, Sea Salt, Citric Acid, DL-Methionine, L-Lysine Hydrochloride, Potassium Chloride, Chondroitin, Glucosamine, Milk Thistle, Vitamin C, Rosemary Extract	All Life Stages (Dog)
Diet 10	Duck Meal, Rice, Salmon Meat, Soy Dietary Fiber (Defatted Soybean Meal), Oats, Wheat Germ, Emulsifier, Refined Chicken Oil, Alpha Corn, Calcium Carbonate, Coconut Powder, Fucoidan, Alginate, Millet Seaweed, Silicate Agent, Chlorella, Spirulina, Plant-Based Collagen, Natural Flavor (Hydrolyzed Chicken Protein Powder), Banana Powder, Lecithin, Vitamin and Mineral Premix, Tocopherol, Ascorbic Acid, Chondroitin, L-Carnitine, Monascus Red Pigment, Yucca Extract, Rosemary Extract, Acidity Regulator, Potassium Sorbate, Purified Water	All Life Stages (Dog)

Diet 11	Brown Rice, White Rice, Oats, Barley, Sweet Potato, Potato, Carrot, Pumpkin, Cabbage, Broccoli, Spinach, Beet, Shiitake Mushroom, Lotus Root, Chickpeas, Hydrolyzed Soy Protein, Dicalcium Phosphate, Monocalcium Phosphate, Vitamins (A, B12, C, D, E), Natural Tocopherols	All Life Stages (Dog)
Diet 12	Animal Protein Premix (Duck, Chicken), Rice, Plant Protein, Corn, Refined Chicken Oil, Sweet Potato, Brown Rice, Sunflower Meal, Oats, Beet Pulp, Barley, Flaxseed, Sesame Meal, Calcium Carbonate, Monocalcium Phosphate, Choline Chloride, Sunflower Seeds, Bacillus subtilis, Clostridium butyricum, Yucca Extract, DL-Methionine, L-Lysine, Sea Salt, Mung Beans, Buckwheat, Inulin, Vitamin Premix (Vitamin A, D3, etc.), Trace Mineral Premix (Iron, Copper, etc.), Carrot, Pumpkin, Biotite, Pumpkin Seeds, Vitamin E, Vitamin C	All Life Stages (Dog)
Diet 13	Corn Gluten Meal, Salmon Meal, Corn, Rice, Chicken Oil, Chicken, Brown Rice, Oats, Sunflower, Barley, Beet Pulp, Natural Flavor Enhancer, Sesame Meal, Flaxseed, Dicalcium Phosphate, Salmon Oil, Calcium Carbonate, Sunflower Seeds, Choline Chloride, Cellulose, Mung Beans, Buckwheat, Sweet Potato, Carrot, Pumpkin, Vitamin Premix (A, D3, E, B1, B2, Pantothenic Acid, Niacin, Folic Acid, Biotin, B12), Mineral Premix (Iron, Copper, Manganese, Zinc, Iodine, Cobalt, Selenium), L-Lysine Hydrochloride, DL-Methionine, Yucca Extract, Pumpkin Seeds, Salt, Rosemary Extract, Vitamin C	All Life Stages (Dog)
Diet 14	Lamb Meal, Grains (Corn, Rice), Meals (Soybean Meal), Hydrolyzed Chicken Meal (Natural Flavor Enhancer), Beet Pulp, Chicken Fat, Banana Powder, Brewer's Yeast, DL-Methionine, Vitamin Premix (Vitamins A, B1, B2, B3, B5, B6, B12, C, D3, E, Folic Acid, Biotin), Mineral Premix (Iron, Zinc, Manganese, Copper, Calcium, Cobalt, Selenium), Fenugreek Extract, Yucca Extract, Protease, Berry Mix (Maqui Berry, Acai Berry, Aronia), Natural Antioxidants (Rosemary, Vitamin E, Citric Acid, Flavonoids)	All Life Stages (Dog)
Diet 15	Animal Protein (Salmon Meal, Duck Meal, Hydrolyzed Salmon Meal, Hydrolyzed Duck Meal), Rice, Corn Gluten, Brown Rice, Oats, Refined Chicken Fat, Barley, Beet Pulp, Sesame Meal, Hydrolyzed Protein, Sunflower Meal, Flaxseed, Corn, Sunflower Seed, Mung Bean, Buckwheat, Sweet Potato, Carrot, Pumpkin, Pumpkin Seed, Mixed Phosphate, Turmeric Powder, Vitamin E, Yucca Extract, Vitamin Premix (A, D3, E, B1, B2, B6, B12, Calcium Pantothenate, Niacin, Biotin, Folic Acid), Mineral Premix (Iron, Copper, Manganese, Zinc, Iodine, Cobalt, Selenium), Choline Chloride, Sea Salt, Chicory Extract, Fructooligosaccharides, Calcium Carbonate, Vegetable Mix (Mango, Strawberry, Orange, Banana, Apple, Beet, Broccoli, Tomato), Milk Thistle, DL-Methionine, L-Lysine Hydrochloride, Vitamin C, Chondroitin, Glucosamine, Probiotics (Lactobacillus plantarum, Lactobacillus acidophilus, Lactobacillus reuteri), Rosemary Extract	All Life Stages (Dog)
Diet 16	Hyrolyzed shrimp, Rice, Hydrolyzed concentrated protein, Mixed animal fats, Beet pulp, Flaxseed, Dicalcium phosphate, Salt, Soybean oil, Coconut, Chondroitin, Vitamin C, Trace mineral premix (Iron, Manganese, Zinc, Copper, Iodine, Selenium, Cobalt), Choline chloride, Dried yeast, Beneficial bacteria (Lactobacillus plantarum, Bacillus subtilis), Taurine, Fructooligosaccharides, Vitamin premix (A, D3, E, B1, B2, B12, biotin, folic acid), Natural vitamin E, Powdered omega-3, Peptide selenium, Herbal extracts (anise, oregano), L-carnitine, Natural antioxidants, Green-lipped mussel, Fruit and	All Life Stages (Dog)

	vegetable extracts (blueberry, strawberry, orange, mango, apple, banana, tomato, broccoli, parsley), Yucca extract, MSM, Grapefruit seed extract	
Diet 17	Chicken, Cod, Sweet potato starch, Egg powder, Hydrolyzed chicken, Chicken fat, Sweet potato, Lentils, Chicken liver, Yeast extract, Blueberry, Cranberry, Carrot, Psyllium dietary fiber, Chicory extract, Processed fiber feed (Opticell), Yucca extract, Brewer's yeast, DL-methionine, Choline chloride, Sodium acid pyrophosphate, Potassium chloride, Carob, Vitamin and mineral premix, Milk thistle extract, Glucosamine, Mucopolysaccharide, L-carnitine, L-lysine, Probiotics, Complex green tea probiotic powder, Rosemary, Tocopherols	All Life Stages (Dog)
Diet 18	Hydrolyzed salmon, Brown Rice, Duck meal, Soybean meal, Chicken oil, Mung beans, Flaxseed, Whey powder, Krill, Oats, Beet pulp, Red ginseng dietary fiber, Bacon, Monocalcium phosphate, Turmeric, Mineral premix, Vitamin premix, Green-lipped mussel powder, Yucca extract, Fish oil, Sea salt, Fructooligosaccharides, Taurine, Inuliin, Methionine, Glucosamine, Concentrated red giinseng extract powder, Seaweed calcium, Red ginseng powder, probiotic blend	All Life Stages (Dog)
Diet 19	Grains (Genetically Modified Corn), Soybean Meal, Chicken Meal, Cottonseed Meal, Animal Fat, Beet Pulp, Limestone, Hydrolyzed Chicken, Dicalcium Phosphate, Refined Salt, Minerals, Vitamins, Lecithin, Lysine Sulfate, Choline Powder, Natural Antioxidant (Rosemary Extract), Chlorella	All Life Stages (Dog)
Diet 20	Lamb Meal, Lentils, Tapioca Starch, Peas, Banana Powder, Beet Pulp, Skim Milk Powder, Cheese Powder, Mixed Compound Feed, Yeast Culture, Crude Fiber, Monocalcium Phosphate, Alfalfa, Premix, Illite, Taurine, Amino Acid Mix, Parsley, Kelp, Propolis, Mealworm, Potato, Sweet Potato	All Life Stages (Dog)
Diet 21	Duck Meal, Soybean Meal, Tapioca Starch, Salmon Oil, Propylene Glycol, Kidney Beans, Cellulose, Hydrolyzed Chicken, Pollock Powder, Red Ginseng Powder, Hydrolyzed Salmon, Green Lipped Mussel Powder, Glucosamine, Coenzyme Q10, Broccoli Powder, Carrot, Parsley, Sweet Potato Starch, Pumpkin Powder, Yucca Extract (Root), Prebiotics (FOS), Astaxanthin, Banana, Apple, Spinach, Kale, Carrot, Napa Cabbage, Broccoli, Vitamin Premix (A, B12, D, E), Mineral Premix (Zinc, Manganese), Amino Acid Premix (DL-Methionine, L-Lysine), Sodium Propionate	All Life Stages (Dog)
Diet 22	Beef, Duck, Rice Flour, Pea Protein Powder, Potato Starch, Beet Pulp, Tapioca, Chicken Oil, Chicory Pulp, Pollock Powder, Sweet Potato, Protein Hydrolysate, Pumpkin, Salmon Protein Powder, Kelp Meal, Hemp Seed, Fructooligosaccharide, Fish Oil, Flaxseed	All Life Stages (Dog)
Diet 23	Corn Gluten Meal, Salmon Meal, Refined Chicken Fat, Rice, Chicken, Corn, Brown Rice, Oats, Sunflower Meal, Barley, Beet Pulp, Natural Flavoring, Sesame Meal, Flaxseed, Mixed Phosphate, Salmon Oil, Sunflower Seed, Choline Chloride, Cellulose, Mung Bean, Buckwheat, Sweet Potato, Calcium Carbonate, Maifanite, Carrot, Pumpkin, Vitamin Premix (Vitamin A, Choline, Vitamin D3, Vitamin E, Folic Acid, Biotin), Mineral Premix (Calcium, Iron, Zinc, Manganese, Copper, Iodine, Cobalt, Selenium), L-Lysine Hydrochloride, DL-Methionine, Yucca Extract, Pumpkin Seed, Salt, Rosemary Extract, Potassium Chloride	All Life Stages (Dog)

Diet 24	Chicken Meal, Rice, Chicken, Chicken Oil, Brown Rice, Oats, Beet Pulp, Protein Hydrolysate (Flavoring Agent), Barley, Rice, Sesame Meal, Sunflower Meal, Flaxseed, Sunflower Seed, Choline Chloride, Mung Bean, Buckwheat, Sweet Potato, Carrot, Pumpkin, DL-Methionine, Monodicalcium Phosphate (MDCP), Potassium Chloride, Inulin, Maifanite, Pumpkin Seed, L-Lysine Sulfate, Yucca Extract, Refined Salt, Beneficial Yeast [Brewer's Yeast (Saccharomyces cerevisiae)], Vitamin Premix (Vitamin A, Vitamin D3, Vitamin E, Vitamin B1, B2, B6, B12, Biotin, Pantothenic Acid, Folic Acid, Niacin), Trace Mineral Premix (Iron, Copper, Zinc, Selenium, Manganese, Iodine, Cobalt), Probiotics (Lactobacillus plantarum), Vitamin E, Vegetable Mix (Broccoli, Parsley, Banana, Apple, Orange, Mango, Strawberry), Rosemary Extract, Vitamin C (L-Ascorbic Acid)	All Life Stages (Dog)
Diet 25	Chicken, Peas, Grains (Corn Grits), Corn Gluten, Flaxseed, Beet Dietary Fiber, Chicken Liver, Concentrated Soy Protein, Oat Dietary Fiber, Dicalcium Phosphate, Chickpeas, Brewer's Yeast, Potassium Chloride, Canola Oil, Calcium Carbonate, Refined Salt, DL-Methionine, L-Lysine, Choline Chloride, Trace Mineral Premix, Vitamin Premix, L-Carnitine, Taurine, Tocopherols, Yeast Extract, Rosemary Extract, Green Tea Extract	All Life Stages (Dog)
Diet 26	Purified Water, Chicken, Pumpkin, Tapioca Starch, Tricalcium Phosphate, Guar Gum, Vitamins (Choline Chloride, Vitamin E, Niacin, Vitamin A, Calcium Pantothenate, Vitamin B2, B6, Biotin, Vitamin B12, Vitamin D3, Vitamin K3, Folic Acid), Minerals (Potassium Chloride, Ferrous Sulfate, Zinc Oxide, Manganese Sulfate, Dicalcium Phosphate, Potassium Iodide, Copper Proteinate, Sodium Selenite, Sodium Chloride, Magnesium Oxide)	All Life Stages (Dog)
Diet 27	Chicken, Sweet Potato, Chicken Broth, Sweet Potato Starch	All Life Stages (Dog)
Diet 28	Chicken, Carrot, Potato, Broccoli, Isolated Soy Protein, Pollock, Pumpkin, Probiotics (Lactobacillus plantarum), Choline Chloride, Green Lipped Mussel, Vitamin Premix (Vitamin D, Vitamin C), Mineral Premix (Iron, Copper), Calcium Carbonate	All Life Stages (Dog)
Diet 29	Chicken, Cabbage, Sweet Potato, Pumpkin, Carrot, Broccoli, Tricalcium Phosphate, Choline Bitartrate, Magnesium Chloride, Zinc Oxide, Vitamin E, Ammonium Ferric Citrate, Niacinamide, Calcium Pantothenate, Copper Gluconate, Vitamin B2, Manganese Sulfate, Vitamin A, Vitamin B1, B6, Potassium Iodide, Selenium, Vitamin B9, B12, D3, Bilberry Concentrate Powder	All Life Stages (Dog)
Diet 30	Chicken Breast, Chicken Heart, Chicken Liver, Calcium Carbonate, Choline Chloride, Zinc, Copper, Magnesium, Manganese, Iron, Kelp Powder, Refined Salt, Vitamin B1, B2, B3, B5, B6, B9, B12, E, D3	All Life Stages (Dog)
Diet 31	Beef, Sweet Potato, Oats, Carrot, King Oyster Mushroom, Kale, Supplement (Dicalcium Phosphate, Potassium Chloride, Salt, Calcium Carbonate, Choline, Taurine, Zinc, Magnesium, Iron Proteinate, Vitamin E, Garbanzo Beans, Copper, Manganese, Vitamin B12, Niacin, Selenium, Riboflavin, Calcium Pantothenate, Thiamine, Vitamin A, D, Calcium Iodate, Vitamin B6, Folic Acid), Apple, Chia Seeds, Cod Liver Oil, Sunflower Oil	All Life Stages (Dog)
Diet 32	Turkey Meat, Turkey Meal, Tapioca, Peas, Lentils, Chickpeas, Canola Oil, Coconut Oil, Natural Flavor, Dicalcium Phosphate, Sodium Chloride, Potassium Chloride, Dried Chicory Root, Choline Chloride, Marine Microalgae Oil, Vitamin Premix (Vitamin A, D3, E, Niacin, L-Ascorbyl-2-Polyphosphate, Thiamine Mononitrate, d-Calcium Pantothenate, Riboflavin,	All Life Stages (Dog)

	Pyridoxine Hydrochloride, Beta-Carotene, Folic Acid, Biotin, Vitamin B12), Trace Mineral Premix (Zinc Proteinate, Iron Proteinate, Copper Proteinate, Zinc Oxide, Manganese Proteinate, Copper Sulfate, Ferrous Sulfate, Calcium Iodate, Manganese Oxide, Selenium Yeast), Taurine, Dried Rosemary	
Diet 33	Chicken, Chicken Liver, Chicken Bone, Chicken Neck, Green Lipped Mussel, Chicken Heart, Chicken Cartilage, Parsley, Apple Pomace, Chicory Inulin, Minerals (Dipotassium Phosphate, Magnesium Sulfate, Zinc Amino Acid Complex, Iron Amino Acid Complex, Copper Amino Acid Complex, Manganese Amino Acid Complex, Dried Yeast [Selenium]), Seaweed Powder, Refined Salt, Citric Acid, Mixed Tocopherols, Vitamin Premix (Vitamin E, B1, B2, B6, D3, Folic Acid)	All Life Stages (Dog)
Diet 34	Water, Chicken, Pork Liver, Brown Rice, Wheat Flour, Carrot, Rice Starch, Potato Starch, Beet Pulp, Egg, Tricalcium Phosphate, Potato, Dextrose, Peas, Hydrolyzed Chicken Flavor, Spinach, Pork Plasma, Soybean Oil, Potassium Chloride, Guar Gum, Fish Oil, Sodium Pyrophosphate, Disodium Phosphate, Sodium Hexametaphosphate, Choline Chloride, Vitamin E, Vitamin C, Trace Mineral Premix (Ferrous Sulfate, Zinc Oxide), Calcium Carbonate, Dicalcium Phosphate, L-Lysine, Magnesium Oxide, Taurine, Caramel Color, Beta-Carotene	All Life Stages (Dog)
Diet 35	Animal Protein (Duck, Chicken, Lamb), Rice, Refined Chicken Fat, Hydrolyzed Chicken Meal (Natural Flavoring), Wheat Bran, Beet Pulp, Peas, Chickpeas, Lentils, Sweet Potato, Hydrolyzed Salmon, Protease (Proteolytic Enzyme), Fruit & Vegetable Mix (Tomato, Beet, Broccoli, Banana, Apple, Orange, Mango, Carrot), Cellulose (Purified Fiber), Refined Fish Oil, DL-Methionine, Vitamin C, Brewer's Yeast, Fenugreek Extract, Yucca Extract, Dicalcium Phosphate, Calcium Carbonate, Potassium Chloride, Vitamin Premix (Vitamin A, D3, E, B1, B2, B6, B12, Biotin, Folic Acid, Pantothenic Acid, Niacin), Trace Mineral Premix (Iron, Zinc, Manganese, Copper, Iodine, Cobalt, Selenium), Natural Antioxidants (Rosemary Extract, Vitamin E, Citric Acid, Flavonoids)	All Life Stages (Dog)
Diet 36	Chicken, Chicken Meal, Lentils, Peas, Chickpeas, Chicken Fat, Banana Powder, Sweet Potato Powder, Beet Pulp, Egg Powder, Hydrolyzed Chicken Meal, Hydrolyzed Salmon Meal, Flaxseed, Refined Salt, Brewer's Yeast, Monocalcium Phosphate, Vitamin Premix (Vitamin A, B1, B2, B3, B5, B6, B12, C, D3, E, Folic Acid, Biotin), Trace Mineral Premix (Ferrous Sulfate, Zinc Sulfate, Manganese Sulfate, Copper Sulfate, Calcium Iodate, Cobalt Sulfate), Organic Minerals (Manganese, Iron, Zinc, Copper, Cobalt, Iodine, Selenium), Curcumin, Butyric Acid, L-Tryptophan, L-Carnitine, Glucosamine, Chondroitin (Mucopolysaccharides), Protease (Proteolytic Enzyme), Yucca Extract, Probiotics, Beta-Carotene, Prebiotics (Inulin, Fructooligosaccharide), Chitosan, Acai Berry, Aronia, Natural Antioxidants (Rosemary, Vitamin E, C, Citric Acid, Flavonoids)	All Life Stages (Dog)
Diet 37	Hydrolyzed Mealworm, Brown Rice, Sweet Potato, Mung Bean, Sunflower Seed, Whole Barley, Peas, Whole Flaxseed, Beet Pulp, Krill Oil, Calcium Phosphate, Yucca Extract, Dried Krill, Fructooligosaccharide, Methionine, Sea Salt, Taurine, Hydrolyzed Green Lipped Mussel Complex, Fucoidan (Seaweed Extract), Glucosamine, MSM (Methylsulfonylmethane), L-Carnitine, Sea Buckthorn Berry, Evening Primrose Seed, Coriander, Carrot, Spinach, Ascorbic Acid, Beneficial Bacteria Blend,	All Life Stages (Dog)

	Dried Probiotics, Vitamin Premix (Vitamin C, E, A, D3, B1, B2, B6, B12, Biotin, Folic Acid, Pantothenic Acid, Niacin, Choline), Mineral Premix (Iron, Zinc, Copper, Selenium, Iodine, Manganese, Potassium)	
Diet 38	Sweet Potato, Whole Barley, Brown Rice, Hydrolyzed Soy Protein, Soy Powder, Sunflower Seed Powder, Whole Flaxseed, Peas, Canola Oil, Methionine, Mung Bean, Whole Oats, Sea Salt, Calcium Phosphate, Yucca Schidigera Extract, Fructooligosaccharide, Inulin, Fenugreek Seed, Sea Buckthorn Berry, Evening Primrose Seed, Carrot, Coriander, Spinach, Beneficial Bacteria Blend, Blueberry, Fucoidan (Seaweed Extract), Vitamins (Vitamin C, E, A, D3, B1, B2, B6, B12, Biotin, Folic Acid, Pantothenic Acid, Niacin), Minerals (Iron, Zinc, Copper, Selenium, Iodine, Manganese)	All Life Stages (Dog)
Diet 39	Hydrolyzed Salmon, Whole Barley, Brown Rice, Mung Bean, Sunflower Seed, Sweet Potato, Peas, Potato, Chicken Fat, Whole Flaxseed, Fish Meal, Fish Oil, Dried Krill, Beet Pulp, Vitamin Premix, Mineral Premix, Yucca Schidigera Extract, Sea Salt, Whole Oats, Fructooligosaccharide, DL-Methionine, Red Ginseng, Hydrolyzed Green Lipped Mussel Complex, Sea Buckthorn Berry, Evening Primrose Seed, Coriander, Carrot, Spinach, Glucosamine, Ascorbic Acid, Beneficial Bacteria Blend, Calcium Phosphate, Fucoidan, Dried Probiotics	Senior (Dog)
Diet 40	Duck Meat, Duck Liver, Carrot, Broccoli, Blueberry, Kale, Duck Heart, Canola Oil, Monocalcium Phosphate, Eggshell Powder, Refined Salt, Tomato Powder, Potassium Chloride, Choline Chloride, DL-Methionine, Fish Oil (Omega-3 Oil), Green Tea Extract, Rosemary Extract, Taurine, Natural Tocopherols, Chicory Inulin, Red Yeast Rice Powder, Trace Mineral Premix, Vitamin Premix, Seaweed Powder, Yeast Extract, L-Carnitine	Adult (Dog)
Diet 41	Chicken Meal, Corn, Probiotics (Bacillus subtilis), Protease (Enzyme), Fenugreek, L-Carnitine, CLA (Conjugated Linoleic Acid), Chicken Oil, Rice, Hydrolyzed Chicken, Black Soldier Fly Powder, Beet Pulp, Flaxseed, Brewer's Yeast, Salt, L-Lysine, DL-Methionine, L-Threonine, Tomato, Apple, Banana, Sugar Beet, Orange, Broccoli, Strawberry, Mango, Parsley, Fructooligosaccharide, Mannan Oligosaccharide, Glucosamine, MSM (Methylsulfonylmethane), Cellulose, Propolis, Yucca Extract, Chelated Minerals (Iron, Copper, Zinc, Selenium, Manganese, Cobalt), Natural Antioxidants (Tocopherols), Rosemary Oil, Vitamin Supplement (Vitamin A, D, E, K, B1, B2, B6, B5, B12, C, Biotin, Folic Acid, Niacin), Choline Chloride, Potassium Chloride	All Life Stages (Dog)
Diet 42	Chicken Meal, Grains, Plant-Based Proteins, Hydrolyzed Protein (Chicken), Meat Meal, Chicken Oil, Beet Pulp, Brewer's Yeast, L-Lysine, DL-Methionine, L-Threonine, Propolis, Fructooligosaccharide, Yucca Extract, Natural Antioxidants (Tocopherols, Rosemary Oil), Vitamin Supplement (Vitamin A, D, E, K, B1, B2, B6, B5, B12, C, Biotin [B8], Folic Acid [B9], Niacin [B3]), Choline Chloride, Mineral Supplement (Iron, Copper, Zinc, Selenium, Manganese, Iodine, Cobalt)	All Life Stages (Dog)
Diet 43	Duck Meal, Soybean Meal, Tapioca Starch, Refined Chicken Fat, Hydrolyzed Chicken, Hydrolyzed Salmon, Beet Pulp, Flaxseed, Fructooligosaccharide, Sweet Potato, Carrot, Pumpkin, Broccoli, Parsley, Yucca Extract (Root), Taurine, L-Carnitine, Garcinia Cambogia Extract, Green Lipped Mussel, Vitamin Premix (Vitamin A, B12, D, E), Mineral Premix (Calcium, Iron, Zinc), Amino Acid Premix (DL-Methionine, L-Lysine)	All Life Stages (Dog)

Diet 44	Animal Protein Premix (Chicken, Beef), Corn Gluten Meal, Brown Rice, Barley, Chicken Oil, Vegetable Glycerin, Rice, Chicken Liver, Sunflower Meal, Sesame Meal, Oats, Puffed Rice, Beet Pulp, Flaxseed, Mung Bean, Fructooligosaccharide, Sunflower Seed, Coconut Powder, Turmeric, Honey, Buckwheat, Sweet Potato, Calcium Phosphate, Ascorbic Acid, Carrot, Pumpkin, Lysine, Methionine, Yucca Extract, Pumpkin Seed, Tocopherols, Rosemary Extract	All Life Stages (Dog)
Diet 45	Duck Meal, Soybean Meal, Pinto Beans, Tapioca Starch, Inactive Dried Yeast, Hydrolyzed Duck, Refined Chicken Fat, Dandelion Extract, Licorice Extract, TF-343 (Saururus Chinensis, Honeysuckle, Balloon Flower Root), Cellulose, Sodium Hexametaphosphate (SHMP), Fructooligosaccharide, Probiotics (Bacillus coagulans), Balloon Flower Root Extract Powder, Vitamin Premix (Vitamin A, B12, D, E), Mineral Premix (Zinc, Manganese), Amino Acid Premix (DL-Methionine, L-Lysine)	All Life Stages (Dog)
Diet 46	Salmon, Oats, Sweet Potato, Pumpkin, Carrot, Apple, Red Cabbage, Celery, Vitamin & Mineral Premix, Amino Mix, Eggshell Powder	All Life Stages (Dog)
Diet 47	Hydrolyzed Duck, Sweet Potato, Potato, Sunflower Seed, Mung Bean, Soybean Meal, Flaxseed, Peas, Beet Pulp, Fish Oil, Dicalcium Phosphate, Mineral Premix, Vitamin Premix, Yucca Extract, Fructooligosaccharide, Methionine, Sea Salt, Taurine, Hydrolyzed Green Lipped Mussel Complex, Red Ginseng, L-Carnitine, Ascorbic Acid, Glucosamine, Sea Buckthorn Berry, Evening Primrose Seed, Coriander, Carrot, Spinach, Beneficial Bacteria Blend	All Life Stages (Dog)
Diet 48	Corn Protein, Rice, Salmon Protein, Chicken, Corn, Chicken Oil, Brown Rice, Oats, Natural Flavoring, Barley, Sesame Meal, Beet Pulp, Sunflower Meal, Flaxseed, Mixed Phosphate, Salmon Oil, Cellulose, Limestone, Sunflower Seed, Mung Bean, Buckwheat, Sweet Potato, Maifanite, Choline Chloride, Carrot, Pumpkin, Pumpkin Seed, Yucca Extract, Sea Salt, Taurine, L-Lysine, Arginine, DL-Methionine, L-Carnitine, Minerals (Calcium, Iron, Zinc, Manganese, Copper, Iodine, Cobalt, Selenium), Vitamins (Vitamin A, Choline, D3, E, Niacin, Pantothenic Acid, B2, B6, B12, K, Folic Acid, Biotin)	Adult (Dog)
Diet 49	Corn Gluten Meal, Salmon Meal, Corn, Rice, Chicken Oil, Chicken, Brown Rice, Oats, Sunflower Meal, Barley, Beet Pulp, Natural Flavoring, Sesame Meal, Flaxseed, Mixed Phosphate, Salmon Oil, Calcium Carbonate, Sunflower Seed, Choline Chloride, Cellulose, Mung Bean, Buckwheat, Sweet Potato, Carrot, Pumpkin, Vitamin Premix (A, D3, E, B1, B2, Pantothenic Acid, Niacin, B6, Folic Acid, Biotin, B12), Mineral Premix (Iron, Copper, Manganese, Zinc, Iodine, Cobalt, Selenium), L-Lysine Hydrochloride, DL-Methionine, Yucca Extract, Pumpkin Seed, Salt, Rosemary Extract, Vitamin C	Senior (Dog)
Diet 50	Rice, Sweet Potato, Chicken, Chicken Oil, Fish Fillet, Hydrolyzed Chicken, Beet Pulp, Salmon Oil, Minerals (Ferrous Sulfate, Copper Sulfate, Zinc Sulfate, Manganese Sulfate, Calcium Iodate, Sodium Selenite), Brewer's Yeast, Chicory, Taurine, Broccoli, Spinach, Cranberry, Blueberry, Herbal Extracts (Yucca Extract, Rosemary Extract, Grape Seed Extract, Turmeric Extract, Grapefruit Extract, Clove Extract), L-Carnitine, Glucosamine, Green Tea Extract	All Life Stages (Dog)
Diet 51	Grains, Chicken Meal, Oilseed Meals, Bran, Mixed Animal Fats, Chicken Oil, Hydrolyzed Chicken Meal (Natural Flavoring), Refined Salt, L-Tryptophan, Vitamin Premix (A, B1, B2, B3, B5, B6, B12, C, D3, E, Folic Acid, Biotin), Mineral Premix (Iron,	Adult (Dog)

	Zinc, Manganese, Copper, Calcium, Cobalt, Selenium), Choline Chloride, Vitamin E, Fenugreek Extract, Yucca Extract, Natural Antioxidants (Rosemary, Vitamin E, Citric Acid, Flavonoids)	
Diet 52	Chicken, Chicken Meal, Chickpeas, Lentils, Peas, Sweet Potato Powder, Banana Powder, Chicken Oil, Hydrolyzed Chicken Meal (Natural Flavoring), Egg Powder, Hydrolyzed Salmon, Flaxseed, Beet Pulp, Brewer's Yeast, Probiotics (Bacillus subtilis, Bacillus licheniformis, Aspergillus oryzae, Lactobacillus acidophilus, Streptococcus thermophilus, Saccharomyces cerevisiae), Prebiotics (Inulin, Fructooligosaccharide), Curcumin, Butyric Acid, Glucosamine, Chondroitin (Mucopolysaccharides), Chitosan, L-Carnitine, Herbal Extract Blend (Angelica, Cnidium, White Peony Root, Rehmannia Root), Beta-Carotene, Calcium Carbonate, Dicalcium Phosphate, DL-Methionine, Potassium Chloride, Vitamin Premix (Vitamin A, B1, B2, B3, B5, B6, B12, C, D3, E, Folic Acid, Biotin), Mineral Premix (Iron, Zinc, Manganese, Copper, Calcium, Cobalt, Selenium), Organic Minerals (Manganese, Iron, Zinc, Copper, Cobalt, Iodine, Selenium), Fenugreek Extract, Yucca Extract, Natural Antioxidants (Rosemary, Vitamin E, Flavonoids), L-Tryptophan, Protease (Proteolytic Enzyme), Berry Mix (Maqui Berry, Acai Berry, Aronia)	Adult (Dog)
Diet 53	Rice, Meat Meal (Lamb), Oilseed Meals (Hydrolyzed Concentrated Protein), Mixed Animal Fats, Beet Pulp, Soybean Oil, Protein Hydrolysate (Hydrolyzed Chicken, Flavoring), Flaxseed, Dried Brewer's Yeast, Cellulose, Hydrolyzed Krill, Zeolite, Refined Salt, Lecithin, Vitamin Premix, Trace Mineral Premix, Chicory Extract, Vitamin C, Yucca Extract, L-Carnitine, Fruit and Vegetable Extracts (Rosemary, Quercetin, Catechin Extract, Tomato, Broccoli, Parsley, Strawberry, Banana, Orange, Mango, Apple), Taurine, MSM (Methylsulfonylmethane), Vitamin E, Zinc Peptide, Organic Selenium, Grapefruit Seed Extract	Senior (Dog)
Diet 54	Hydrolyzed Chicken, Barley, Brown Rice, Chicken Meal, Sunflower Meal, Soybean Meal, Flaxseed, Sweet Potato, Chicken Oil, Red Ginseng Dietary Fiber, Dicalcium Phosphate, Steamed Ginseng Concentrate Powder, Mung Bean, Oats, Vitamin Premix, Mineral Premix, Yucca Extract, Alginate, Beneficial Bacteria Blend, Inulin, Sea Salt, Methionine, Alpha-Tocopherol, Red Ginseng Powder, Ascorbic Acid, Evening Primrose Seed, Sea Buckthorn Berry, Spinach, Coriander, Carrot	All Life Stages (Dog)
Diet 55	Corn, Wheat, Corn Gluten, Chicken Meal, Soy Flour, Refined Chicken Oil, Calcium Carbonate, Salt, Palatability Enhancer, Mineral Premix (Iron, Copper, Zinc, Manganese, Cobalt, Iodine, Selenium), Calcium Phosphate, Vitamin Premix (Vitamin A, B12, Biotin, Pantothenic Acid, Folic Acid, Niacin)	Adult (Dog)
Diet 56	Corn Protein, Rice, Salmon Protein, Chicken, Corn, Chicken Oil, Brown Rice, Oats, Natural Flavoring, Barley, Sesame Meal, Beet Pulp, Sunflower Meal, Flaxseed, Mixed Phosphate, Salmon Oil, Cellulose, Limestone, Sunflower Seed, Mung Bean, Buckwheat, Sweet Potato, Maifanite, Choline Chloride, Carrot, Pumpkin, Pumpkin Seed, Yucca Extract, Sea Salt, Taurine, L-Lysine, Arginine, DL-Methionine, L-Carnitine, Minerals (Calcium, Iron, Zinc, Manganese, Copper, Iodine, Cobalt, Selenium), Vitamins (Vitamin A, Choline, D3, E, Niacin, Pantothenic Acid, B2, B6, B12, K, Folic Acid, Biotin)	Adult (Dog)
Diet 57	Hydrolyzed Salmon, Lamb, Brown Rice, Barley, Sweet Potato, Sunflower Meal, Chicken Fat, Flaxseed, Dicalcium Phosphate, Fish Oil, Inulin, Fenugreek Seed Extract, Oats, Fructooligosaccharide, Sea Buckthorn Berry, Sea Salt, DL-Methionine, Sodium	Senior (Dog)

	Alginate, Taurine, Vitamin Premix (Vitamin A, D3, C, E, B1, B2, B6, B12, Biotin, Pantothenic Acid, Folic Acid, Niacin), Mineral Premix (Iron, Copper, Zinc, Selenium, Manganese, Iodine), Microorganism Blend, Carrot, Coriander, Spinach, Evening Primrose Seed, Choline Chloride, Shark Cartilage Mucopolysaccharide (Chondroitin), Glucosamine, Ascorbic Acid	
Diet 58	Beef, Chickpeas, Lentils, Pea Protein, Tapioca Starch, Pea Starch, Flaxseed, Beet Dietary Fiber, Beef Fat, Canola Oil, Chicken Liver, Dicalcium Phosphate, Brewer's Yeast, Egg Powder, Calcium Carbonate, Potassium Chloride, Refined Salt, Apple, Carrot, Pumpkin, Blueberry, Fish Oil, Taurine, Trace Mineral Premix, Vitamin Premix, Natural Tocopherols, Rosemary Extract, Green Tea Extract, Chicory Inulin, L-Lysine, Glycerin Fatty Acid Ester, Choline Chloride, DL-Methionine, L-Carnitine	Adult (Dog)
Diet 59	Chicken, Peas, Grains (Corn Grits), Corn Gluten, Flaxseed, Beet Dietary Fiber, Dicalcium Phosphate, Chicken Liver, Beef Fat, Tapioca Starch, Canola Oil, Chicken Wing Tips, Brewer's Yeast, Coconut, Potassium Chloride, Turmeric, Calcium Carbonate, Fish Oil, Glucosamine, L-Tyrosine, Lecithin, Blueberry, Acai Berry, Maqui Berry, L-Carnitine, DL-Methionine, L-Lysine, Taurine, Choline Chloride, Trace Mineral Premix, Vitamin Premix, Refined Salt, Rosemary Extract, Green Tea Extract, Tocopherols, Yeast Extract	Senior (Dog)
Diet 60	Ready-to-Eat Food (Chicken, Purified Water, Carrot, Brown Rice, Sweet Potato, Broccoli, Chicken Liver, Egg White, Chickpeas, Cabbage, Kale Leaf, Chicken Fat, Turmeric, Fish Oil, Shiitake Mushroom, Dicalcium Phosphate, Eggshell Powder, Kelp Powder), DL-Methionine, Trace Mineral Premix, Vitamin Premix, L-Carnitine	All Life Stages (Dog)
Diet 61	Skipjack Tuna (White Meat), Purified Water, Potato, Carrot, Pumpkin, Fruit and Vegetable Concentrate (Apple Juice Concentrate, Pear Juice Concentrate, Cabbage Extract, Celery Extract Powder, Broccoli Concentrate Powder), Peas, Cranberry Concentrate, Heat-Treated Lactic Acid Bacteria, Pollock Extract, Concentrated Chicken Broth, Tapioca Starch, Amino Base, Nutrient Fortifier	All Life Stages (Dog)
Diet 62	Purified Water, Beef, Chicken, Carrot, Pumpkin, Tapioca Starch, Flavoring, Whole Egg Powder, Peas, Binders (Carrageenan, Guar Gum, Potassium Chloride), Salt, Sodium Tripolyphosphate, Mineral Premix (Zinc Oxide, Potassium Iodide, etc.), Caramel Color, Vitamin Premix (Vitamin E, B12, A, Beta-Carotene, Folic Acid, etc.), Sodium Acid Pyrophosphate, Tuna Oil, Coenzyme Q10	Adult (Dog)
Diet 63	Purified Water, Chicken, Pork Liver, Canola Oil, Wheat Gluten, Carrot, Red Ginseng Concentrate, Vitamin and Mineral Premix (Calcium, Potassium, Vitamin E, B3, B12, etc.), Carrageenan, Xanthan Gum	Senior (Dog)
Diet 64	Beef (Chuck), Potato, Cauliflower, Carrot, Green Beans, Peas, Beef Liver, Sunflower Oil, Salmon Oil, Dicalcium Phosphate, Coral Calcium, Vitamin E, Vitamin D3, Calcium Iodate, Manganese Sulfate, Magnesium Oxide, Zinc Sulfate	All Life Stages (Dog)
Diet 65	Salmon, Carrot, Broccoli, Bell Pepper, Cabbage, Spinach, Mushroom, Banana, Blueberry, Oatmeal, Hemp Seed, Hemp Seed Oil, Bromelain, Eggshell Powder, Custom Formulated Supplement	Adult (Dog)
Diet 66	Rice, Wheat Flour, Meat Meal (Chicken, Duck), Corn, Animal Fat (Chicken, Duck), Wheat Gluten, Protein Hydrolysate (Chicken, Turkey, Fish), Cornmeal, Beet Pulp, Brewer's Yeast, Soybean Oil, Powdered Cellulose, Zeolite, Refined Salt, Vitamin	Adult (Dog)

	Premix, Calcium Carbonate, DL-Methionine, Monocalcium Phosphate, Glycerin Fatty Acid Ester, Potassium Chloride, Mixed Mineral Premix, Fish Oil, Fructooligosaccharide, Sodium Polyphosphate, Potassium Sorbate, Choline Chloride, L-Lysine, Vitamin E (Antioxidant), L-Tyrosine, Algal Oil (Source of EPA+DHA), Vitamin C, L-Carnitine, Vegetable Oil, Magnesium Oxide, Rosemary Extract	
Diet 67	Chicken Meal, Barley, Pea Fiber, Brown Rice, Whole Corn, Corn Gluten Meal, Powdered Cellulose, Whole Oats, Soybean Meal, Whole Sorghum, Rice, Natural Flavoring (Chicken Liver), Soybean Oil, Peas, Beet Pulp, Flaxseed, Lactic Acid, Pork Liver Flavor, Potassium Chloride, Calcium Carbonate, Refined Salt, Vitamin Supplement (Vitamin E, C), Choline Chloride, L-Lysine, Trace Mineral Premix (Ferrous Sulfate, Zinc Oxide), Taurine, d-Tocopherols, Natural Flavoring, L-Carnitine, Beta-Carotene	Adult (Dog)
Diet 68	Purified Water, Chicken, Beef, Gelling Agents (Guar Gum, Sodium Polyphosphate, Carrageenan Gum, Cassia Gum), Sunflower Oil, Mineral Premix (Calcium Carbonate, Potassium Chloride, Zinc Sulfate, etc.), Amino Acid Premix (DL-Methionine, L-Cysteine Hydrochloride), Vitamin Premix (Vitamin E, B5, B1, B12, B6, etc.), Glucose, Xylose, Glycine, Calcium Disodium EDTA (Antioxidant), Sodium Nitrite (Color Fixative)	Adult (Dog)
Diet 69	Chicken Meal, Soy Powder, Corn, Tapioca, Chicken Fat, Hydrolyzed Chicken, Hydrolyzed Fish, Fish Meal, Refined Salt, Vitamins (Vitamin A, D3, E, C, Niacin, B1, B6, d-Calcium Pantothenate, B2, Vitamin K3 Sodium Bisulfite, Nicotinamide, Folic Acid, Biotin, B12), Minerals (Ferrous Sulfate, Zinc Sulfate, Copper Sulfate, Manganese Sulfate, Potassium Iodide, Sodium Selenite), Choline Chloride, DL-Methionine, Taurine, Dicalcium Phosphate	All Life Stages (Cat)
Diet 70	Animal Protein (Chicken, Salmon, Pollock Meal), Corn Gluten Meal, Chicken Oil, Rice, Pea Protein, Natural Flavoring, Sunflower Meal, Brown Rice, Corn, Oats, Beet Pulp, Barley, Rice, Flaxseed, Sesame Meal, Lignocellulose, Choline Chloride, Mixed Phosphate, Sunflower Seed, L-Lysine Sulfate, DL-Methionine, Yucca Extract, Taurine, Vitamin Premix, Mineral Premix, Mung Bean, Buckwheat, Sweet Potato, Salmon Oil, Carrot, Pumpkin, Refined Salt, Maifanite, Pumpkin Seed, Probiotic (Enterococcus faecalis EF-2001), Vitamin E, Calcium Carbonate, L-Carnitine, Rosemary Extract, Cranberry, Vitamin C	Kitten (Cat)
Diet 71	Chicken, Chicken Meal, Chickpeas, Chicken Fat, Lentils, Peas, Fish Meal, Beet Pulp, Banana Powder, Purified Cellulose, Hydrolyzed Salmon Meal, Hydrolyzed Chicken Meal, Brewer's Yeast, Flaxseed, Soybean Oil, Choline Chloride, Beta-Carotene, Organic Minerals (Manganese, Iron, Zinc, Copper, Cobalt, Iodine, Selenium), Curcumin, Butyric Acid, DL-Methionine, Taurine, Vitamin Premix (Vitamin A, B1, B2, B3, B5, B6, B12, C, D3, E, Folic Acid, Biotin), Trace Mineral Premix (Ferrous Sulfate, Zinc Sulfate, Manganese Sulfate, Copper Sulfate, Calcium Iodate, Cobalt Sulfate), Fenugreek, Probiotics, Prebiotics (Inulin, Fructooligosaccharide), Protease, Yucca Extract, Maqui Berry, Acai Berry, Aronia, Natural Antioxidants (Rosemary, Vitamin E, C, Citric Acid, Flavonoids)	All Life Stages (Cat)
Diet 72	Chicken Meal, Corn, Tuna Meal, Chicken Oil, Plant-Based Proteins, Beet Pulp, Flaxseed, Brewer's Yeast, Salt, Propolis, L-Lysine, DL-Methionine, L-Threonine, Taurine, Mannan Oligosaccharide (MOS), Fructooligosaccharide (FOS), Yucca Extract,	All Life Stages (Cat)

	Natural Antioxidants (Tocopherols, Rosemary Oil), Vitamin Supplement (Vitamin A, D, E, K1, B1, B2, B6, B5, Biotin [B8], Folic Acid [B9], Niacin [B3], B12, C), Choline Chloride, Chelated Minerals (Iron, Copper, Zinc, Selenium, Manganese, Iodine), Mineral Supplement (Iron, Copper, Zinc, Selenium, Manganese, Iodine, Cobalt), Potassium Chloride	
Diet 73	Chicken Meal, Soybean Meal, Brown Rice, Bran (Fresh Rice Bran), Mixed Animal Fats, Fish Meal (Tuna), Beet Pulp, Refined Salt, Hydrolyzed Flavoring (Liquid), Hydrolyzed Flavoring (Powder), Zeolite, Calcium Sulfate, Trace Mineral Premix, Dicalcium Phosphate, Vitamin Premix, Choline Chloride, L-Threonine, Grapefruit Seed Extract	All Life Stages (Cat)
Diet 74	Chicken, Peas, Grains (Corn Grits), Corn Gluten, Beet Dietary Fiber, Flaxseed, Oat Fiber, Chicken Liver, Monocalcium Phosphate, White Fish Meal (Cod), Beef Fat, Brewer’s Yeast, Concentrated Soy Protein, Calcium Carbonate, Potassium Chloride, Canola Oil, Refined Salt, Sodium Acid Pyrophosphate, L-Lysine, Chicory Inulin, DL-Methionine, Choline Chloride, Taurine, L-Carnitine, Trace Mineral Premix, Vitamin Premix, Natural Tocopherols, Rosemary Extract, Green Tea Extract, Yeast Extract	All Life Stages (Cat)
Diet 75	Chicken (Fresh Chicken Meat, Dried Powder [Meat, Liver, Heart, Intestine], Hydrolyzed Protein), Salmon (Fresh Salmon, Salmon Meal), Banana, Sweet Potato, Lentils, Chicken Oil, Green Lipped Mussel, Raw Manuka Honey (UMF+5), Peppermint, Turmeric, Parsley, Cranberry, Blueberry, Strawberry, L-Lysine, DL-Methionine, L-Threonine, Taurine, DHA, L-Carnitine, Tomato, Apple, Sugar Beet, Orange, Broccoli, Mango, Beet Pulp, Flaxseed, Brewer’s Yeast, Fructooligosaccharide, Mannan Oligosaccharide, Yucca Extract, Cellulose, Chelated Minerals (Iron, Copper, Zinc, Selenium, Manganese, Cobalt), Natural Antioxidants (Tocopherols, Rosemary Oil), Dried Kelp, Vitamin Supplement (Vitamin A, D, E, K1, B1, B2, B6, B5, Biotin [B8], Folic Acid [B9], Niacin [B3], B12, C), Choline Chloride, Mineral Supplement (Iron, Copper, Zinc, Selenium, Manganese, Iodine, Cobalt), Potassium Chloride, Lactobacillus plantarum, Bacillus subtilis, Enzyme (Protease), Fenugreek	All Life Stages (Cat)
Diet 76	Chicken, Dried Chicken, Chicken Fat, Fish Meal, Beet Pulp, Chickpeas, Cassava (Manioc), Lentils, Banana, Cellulose, Protein Hydrolysate (Hydrolyzed Chicken Meal), Protein Hydrolysate (Bioflavor), Flaxseed, Peas, Fish Oil, Brewer’s Yeast, Gelatin, Choline Chloride, Extract Blend (Beta-Carotene), Hemp Seed Mix (Coconut Flower Extract Powder, Hemp Seed, Probiotic Fermented Extract Powder [Contains GABA]), Fenugreek Extract, Yucca Extract, Taurine, Inulin/Chicory Extract, Fructooligosaccharide, Vitamin Premix (A, B, etc.), Trace Mineral Premix (Zinc, Copper, etc.), L-Lysine, Probiotic (Lactobacillus plantarum), Antioxidant Blend (Rosemary, Flavonoids), Probiotic Complex, Berries (Maqui Berry, Acai Berry, Aronia), Refined Salt, Alkaline (Bacterial) Protease, Vitamin C, Glucosamine, DL-Methionine, Trace Mineral Premix (Selenium, Zinc, etc.), Mucopolysaccharide (Chondroitin)	All Life Stages (Cat)
Diet 77	Grains, Oilseed Meals, Bran, Poultry By-products, Fish Meal, Flaxseed, Chicken Oil, Refined Salt, Vitamin Supplement (Vitamin A, E), Trace Minerals (Iron, Copper, Zinc, Manganese), Potassium Chloride, Yucca Extract	All Life Stages (Cat)
Diet 78	Chicken Meal, Wheat, Corn Gluten Meal, Chicken Oil, Corn, Wheat Bran, Corn Protein Concentrate, Soybean Meal, Flavoring Agent, Beet Pulp, Banana Powder, Inactive Yeast (Brewer’s Yeast), Fish Meal, Botanical Extract (Carob Tree Extract),	Kitten (Cat)

	Hydrolyzed Chicken Meal, Limestone Powder, Refined Salt, Mixed Minerals (Sodium, Potassium), Chicory, Mixed Compound Feed (L-Lysine Hydrochloride, Yucca Extract, Calcium Carbonate), Potassium Chloride, Trace Mineral Premix (Iron, Copper, Zinc, Manganese, Cobalt, Iodine, Selenium), Taurine, DL-Methionine, Vitamin E, Choline Chloride, Vitamin Premix (Vitamin A, B12, Biotin, Pantothenic Acid, Folic Acid, Niacin, Carnitine), Monodicalcium Phosphate (MDCP)	
Diet 79	Purified Water, Chicken, Wheat Gluten, Tuna, Canola Oil, Red Ginseng Concentrate, Inulin, Vitamin and Mineral Mix, Flavoring Agent, Gelling Agent	Kitten (Cat)
Diet 80	Chicken Breast, Chicken Thigh, Chicken Heart, Chicken Liver, Dicalcium Phosphate, Calcium Carbonate, Salt, Vitamin and Mineral Mix	All Life Stages (Cat)
Diet 81	Meat Meal (Chicken, Duck, Turkey), Whole Corn, Wheat, Bread Crumbs, Fish Extract, Dried Yeast Powder, Bonito Extract, Chicken Powder, Tuna Powder, Mackerel Powder, Animal Fat (Beef), Soybean Meal, Palm Oil, Hydrolyzed Chicken Liver, Fish Meal (with Taurine), Vitamins (A, D3, E, K, B2, B3, B6, B12, Pantothenic Acid, Niacin, Biotin, Folic Acid), Minerals (Iron, Manganese, Copper, Zinc, Iodine, Selenium), Sodium Pyrophosphate, DL-Methionine, Heat-Treated Probiotics (Enterococcus faecalis)	All Life Stages (Cat)
Diet 82	Chicken, Chicken Liver, Chicken Bone, Chicken Neck, Green Lipped Mussel, Chicken Heart, Chicken Cartilage, Chicory Inulin, Apple Pomace, Minerals (Dipotassium Phosphate, Magnesium Sulfate, Zinc Amino Acid Complex, Iron Amino Acid Complex, Copper Amino Acid Complex, Manganese Amino Acid Complex, Sodium Selenite), Seaweed Powder, Refined Salt, Citric Acid, Mixed Tocopherols, DL-Methionine, Vitamin Premix (Choline Chloride, Vitamin B1, B6, Folic Acid, D3), Taurine	All Life Stages (Cat)
Diet 83	Mackerel, Purified Water, Chickpeas, Green Lipped Mussel, DL-Methionine, Minerals (Dipotassium Phosphate, Magnesium Sulfate, Zinc Amino Acid Complex, Manganese Amino Acid Complex, Copper Amino Acid Complex), Vitamin Premix (Choline Chloride, Vitamin E, B1, B3, B6, B2, D3, Folic Acid), Seaweed Powder, Refined Salt, Taurine	All Life Stages (Cat)
Diet 84	Animal Protein (Chicken, Salmon, Duck, Sardine), Rice, Refined Chicken Fat, Hydrolyzed Chicken Meal (Natural Flavoring), Peas, Refined Fish Oil, Chickpeas, Lentils, Sweet Potato, Flaxseed, Cellulose (Purified Fiber), Fruit & Vegetable Mix (Tomato, Beet, Broccoli, Banana, Apple, Orange, Mango, Carrot), DL-Methionine, Taurine, L-Lysine, Vitamin C, Protease, Brewer’s Yeast, Fenugreek Extract, Yucca Extract, Dicalcium Phosphate, Calcium Carbonate, Potassium Chloride, Vitamin Premix (Vitamin A, D3, E, B1, B2, B6, B12, Biotin, Folic Acid, Pantothenic Acid, Niacin), Trace Mineral Premix (Iron, Zinc, Manganese, Copper, Iodine, Cobalt, Selenium), Tannin, Natural Antioxidants (Rosemary Extract, Vitamin E, Citric Acid, Flavonoids), Berry Mix (Maqui Berry, Acai Berry, Aronia)	Adult (Cat)
Diet 85	Chicken, Peas, Pea Protein, Pea Starch, Chicken Fat, White Fish Meal (Cod), Concentrated Soy Protein, Beet Dietary Fiber, Chicken Liver, Monocalcium Phosphate, Egg Powder, Chickpeas, Flaxseed, Brewer’s Yeast, Calcium Carbonate, Potassium Chloride, Sodium Acid Pyrophosphate, Refined Salt, DL-Methionine, Choline Chloride, Fish Oil, Glycerin Fatty Acid Ester,	Adult (Cat)

	Taurine, Chicory Inulin, L-Lysine, Trace Mineral Premix, Vitamin Premix, Natural Tocopherols, Yeast Extract, Rosemary Extract, Green Tea Extract, L-Carnitine	
Diet 86	Animal Protein (Chicken Meal, Salmon Meal), Corn Gluten Meal, Refined Chicken Fat, Rice, Beet Pulp, Protein Hydrolysate, Cellulose, Sunflower Meal, Brown Rice, Oats, Barley, Flaxseed, Corn Gluten Meal, Sesame Meal, Choline Chloride, Corn, DL-Methionine, Salmon Oil, Yucca Extract, L-Lysine Hydrochloride, Sunflower Seed, Potassium Chloride, Vitamin Premix (Vitamin A, D3, E, B1, B2, B6, B12, Calcium Pantothenate, Niacin, Biotin, Folic Acid), Mineral Premix (Iron, Copper, Manganese, Zinc, Iodine, Cobalt, Selenium), Mung Bean, Buckwheat, Sweet Potato, Taurine, Citric Acid, Chicory Extract, Vitamin E, Carrot, Pumpkin, Pumpkin Seed, Sea Salt, Turmeric Powder, Fructooligosaccharide, Mixed Phosphate, Rosemary Extract, Vegetable Mix (Mango, Strawberry, Orange, Banana, Apple, Beet, Broccoli, Tomato), Milk Thistle, Probiotics (Bifidobacterium animalis lactis, Lactobacillus rhamnosus, Lactobacillus plantarum), Vitamin C	Adult (Cat)
Diet 87	Mixed Animal Protein Meal (Chicken), Grains, Chicken Fat, Oilseed Meals, Beet Pulp, Fish Meal, Bran, Shrimp Meal, Lecithin, Glucosamine, Refined Salt, Potassium Chloride, DL-Methionine Hydroxy Analog, Vitamin Premix (Vitamin A, B1), Trace Mineral Premix (Copper, Zinc), Yeast Culture, Choline Chloride, Taurine, Potassium Sorbate, L-Tryptophan, Yucca Extract, Fruit and Vegetable Extracts	Adult (Cat)
Diet 88	Hydrolyzed Chicken, Sweet Potato, Mung Bean, Sunflower Seed, Peas, Whole Flaxseed, Potato, Tuna Powder, Dried Krill, Cheese, Smoked Bluefin Tuna, Beet Pulp, Methionine, Yucca Schidigera Extract, Taurine, Inulin, Sea Salt, Fructooligosaccharide, Alginate, Paprika, Dicalcium Phosphate, Probiotics, Sea Buckthorn Berry, Evening Primrose Seed, Hydrolyzed Salmon, Coriander, Carrot, Spinach, Vitamin and Mineral Premix	Adult (Cat)
Diet 89	Black Soldier Fly Larvae, Pea Protein, Refined Chicken Oil, Beet Pulp, Sweet Potato, Peas, Brewer's Yeast, Sweet Potato (duplicate), Flaxseed, Sunflower Meal, Cellulose, Premix (Glucosamine, MSM, Chondroitin, Green Lipped Mussel, Hyaluronic Acid, Safflower Seed, Turmeric, Hijiki, Milk Thistle, Acanthopanax), Sesame Meal, Sunflower Seed, Carrot, Pumpkin, Mixed Phosphate, Choline Chloride, Mung Bean, Hemp Seed, DL-Methionine, Yucca Extract, L-Lysine Sulfate, Refined Salt, Vitamin Premix, Mineral Premix, Taurine, Vitamin E, Citric Acid, Fructooligosaccharide, Rosemary Extract, Maifanite, Vitamin C	All Life Stages (Cat)
Diet 90	Chicken, Fish, Grains, Soybean Meal, Chicken Oil, Beet Pulp, Calcium Phosphate, Salt, Calcium Chloride, Zinc Oxide, Lysine, Choline, Chitosan, Yucca Extract, Iron, Manganese, Zinc, Cobalt, Selenium, Vitamins (A, D, E, K, B1, B2, B6, B12, Niacin, Biotin, Folic Acid, Pantothenic Acid), Taurine	Adult (Cat)
Diet 91	Corn, Chicken Meal, Corn Protein Concentrate, Wheat, Refined Chicken Oil, Wheat Protein Concentrate, Beet Pulp, Natural Palatability Enhancer, White Fish, Salt, Potassium Chloride, Inulin (Chicory Root Extract), Choline Chloride, L-Lysine, DL-Methionine, Mineral Premix (Iron, Copper, Zinc, Manganese, Cobalt, Iodine, Selenium), Taurine, Vitamin Premix (Vitamin A,	Adult (Cat)

	B12, Biotin, Pantothenic Acid, Folic Acid, Niacin), Vitamin E, Yucca Extract, Vegetable Mix (Tomato, Blueberry, Parsley, Broccoli)	
Diet 92	Hydrolyzed Salmon Protein, Rice, Barley, Oats, Sesame, Sunflower Seed, Buckwheat, Flaxseed, Duck Meal, Taurine, Collagen, Yucca Extract, Vitamin Mix (Vitamin A, D3, E, K3, B1, B2, B5, B6, Folic Acid, Niacin, Biotin), Beet Pulp, Vitamin E	Adult (Cat)
Diet 93	Chicken Meal, Corn, Tuna Meal, Chicken Oil, Plant-Based Proteins, Beet Pulp, Flaxseed, Brewer's Yeast, Salt, L-Lysine, DL-Methionine, L-Threonine, Taurine, Propolis, Fructooligosaccharide, Yucca Extract, Natural Antioxidants (Tocopherols, Rosemary Oil), Vitamin Supplement (Vitamin A, D, E, K, B1, B2, B6, B5, Biotin, Folic Acid, Niacin, B12, C), Choline Chloride, Mineral Supplement (Iron, Copper, Zinc, Selenium, Manganese, Iodine, Cobalt), Potassium Chloride	Adult (Cat)
Diet 94	Chicken, Carrot, Broccoli, Mung Bean, Chicken Liver, Beef Kidney, Sweet Potato, Krill, Calcium Carbonate, Choline Chloride, Taheebo, Taurine, Vitamin Premix (Vitamin C, B12, B6, B1, A, E), Mineral Premix (Calcium, Magnesium, Iron, Zinc)	All Life Stages (Cat)
Diet 95	Wheat, Rice, Meat Meal (Chicken, Duck), Wheat Gluten, Corn, Animal Fat (Chicken, Duck), Animal-Derived Protein (Chicken, Turkey, Fish), Wheat Flour, Powdered Cellulose, Mixed Mineral Premix, Beet Pulp, Soybean Oil, Yeast, Fish Oil, Fructooligosaccharide, Psyllium Dietary Fiber, L-Carnitine, Amino Acid Premix, Vitamin A, D3, Iron, Iodine, Copper, Manganese, Zinc, Selenium, Zeolite, Antioxidant	Adult (Cat)
Diet 96	Animal Protein (Chicken, Turkey, Pork), Corn, Wheat, Corn Gluten Meal, Poultry Fat, Peas, Vitamins, Trace Minerals and Other Additives (Vitamin A, D3, E, Biotin, Copper, Zinc, Manganese, Iodine, Selenium, Taurine, Enterococcus faecium, Ammonium Chloride, Bentonite), Rice, Beet Pulp, Hydrolyzed Animal Protein, Alfalfa Protein Concentrate, Flaxseed, Brewer's Yeast, Apple Fiber, Fava Bean, Refined Salt, Calcium Carbonate, Derived Protein (Cod Protein Enzymatic Hydrolysate Marine Peptides), Dried Carrot, Potassium Chloride, Fructooligosaccharide, Calcium Sulfate, Yucca	Adult (Cat)
